# Effectiveness of SARS-CoV-2 Vaccines for Short- and Long-Term Immunity: A General Overview for the Pandemic Contrast

**DOI:** 10.3390/ijms23158485

**Published:** 2022-07-30

**Authors:** Alessio Danilo Inchingolo, Giuseppina Malcangi, Sabino Ceci, Assunta Patano, Alberto Corriero, Luigi Vimercati, Daniela Azzollini, Grazia Marinelli, Giovanni Coloccia, Fabio Piras, Giuseppe Barile, Vito Settanni, Antonio Mancini, Nicole De Leonardis, Grazia Garofoli, Giulia Palmieri, Ciro Gargiulo Isacco, Biagio Rapone, Arnaldo Scardapane, Luigi Curatoli, Nicola Quaranta, Mario Ribezzi, Maria Massaro, Megan Jones, Ioana Roxana Bordea, Gianluca Martino Tartaglia, Antonio Scarano, Felice Lorusso, Luigi Macchia, Angela Maria Vittoria Larocca, Sergey Khachatur Aityan, Silvio Tafuri, Pasquale Stefanizzi, Giovanni Migliore, Nicola Brienza, Gianna Dipalma, Gianfranco Favia, Francesco Inchingolo

**Affiliations:** 1 Department of Interdisciplinary Medicine, Section of Dental Medicine, University of Bari “Aldo Moro”, 70124 Bari, Italy; ad.inchingolo@libero.it (A.D.I.); giuseppinamalcangi@libero.it (G.M.); s.ceci@studenti.uniba.it (S.C.); assuntapatano@gmail.com (A.P.); luigi.vimercati@uniba.it (L.V.); daniela.azzollini93@gmail.com (D.A.); graziamarinelli@live.it (G.M.); giovanni.coloccia@gmail.com (G.C.); dott.fabio.piras@gmail.com (F.P.); g.barile93@hotmail.it (G.B.); v.settanni@libero.it (V.S.); dr.antonio.mancini@gmail.com (A.M.); nicoledeleonardis@outlook.it (N.D.L.); graziagarofoli@libero.it (G.G.); giuliapalmieri13@gmail.com (G.P.); drciroisacco@gmail.com (C.G.I.); biagiorapone79@gmail.com (B.R.); arnaldo.scardapane@uniba.it (A.S.); nicolaantonioadolfo.quaranta@uniba.it (N.Q.); megan.jones@live.co.uk (M.J.); giannadipalma@tiscali.it (G.D.); gianfranco.favia@uniba.it (G.F.); 2 Unit of Anesthesia and Resuscitation, Department of Emergencies and Organ Transplantations, Aldo Moro University, 70121 Bari, Italy; alberto.corriero@gmail.com (A.C.); mario.ribezzi@libero.it (M.R.); nicola.brienza@uniba.it (N.B.); 3 Department Neurosciences & Sensory Organs & Musculoskeletal System, University of Bari “Aldo Moro”, 70124 Bari, Italy; lcuratoli@icloud.com; 4 Azienda Ospedaliero-Universitaria Consorziale Policlinico di Bari, 70124 Bari, Italy; brimas08@gmail.com; 5 Department of Oral Rehabilitation, Faculty of Dentistry, Iuliu Hațieganu University of Medicine and Pharmacy, 400012 Cluj-Napoca, Romania; roxana.bordea@ymail.com; 6 UOC Maxillo-Facial Surgery and Dentistry, Department of Biomedical, Surgical and Dental Sciences, School of Dentistry, Fondazione IRCCS Ca Granda, Ospedale Maggiore Policlinico, University of Milan, 20100 Milan, Italy; gianluca.tartaglia@unimi.it; 7 Department of Innovative Technologies in Medicine and Dentistry, University of Chieti-Pescara, 66100 Chieti, Italy; a.scarano@unich.it; 8 Department of Emergency and Organ Transplantation (D.E.T.O.), University of Bari Aldo Moro, 70124 Bari, Italy; luigi.macchia@uniba.it; 9 Hygiene Complex Operating Unit, Azienda Ospedaliero-Universitaria Consorziale Policlinico di Bari, Place Giulio Cesare 11 BARI CAP, 70124 Bari, Italy; laroccaangela1@gmail.com; 10 Director Multidisciplinary Research Center, Lincoln University, Oakland, CA 94612, USA; aityan@lincolnuca.edu; 11 Department of Biomedical Science and Human Oncology, University of Bari, 70121 Bari, Italy; silvio.tafuri@uniba.it; 12 Interdisciplinary Department of Medicine, University Hospital of Bari, 70100 Bari, Italy; pasquale.stefanizzi@uniba.it (P.S.); giovanni.migliore@policlinico.ba.it (G.M.)

**Keywords:** MERS, SARS-CoV-2, SARS-CoV-1, COVID-19, antibodies, anti-Spike, vaccines, dentistry, Pfizer, booster

## Abstract

Background: The recent COVID-19 pandemic produced a significant increase in cases and an emergency state was induced worldwide. The current knowledge about the COVID-19 disease concerning diagnoses, patient tracking, the treatment protocol, and vaccines provides a consistent contribution for the primary prevention of the viral infection and decreasing the severity of the SARS-CoV-2 disease. The aim of the present investigation was to produce a general overview about the current findings for the COVID-19 disease, SARS-CoV-2 interaction mechanisms with the host, therapies and vaccines’ immunization findings. Methods: A literature overview was produced in order to evaluate the state-of-art in SARS-CoV-2 diagnoses, prognoses, therapies, and prevention. Results: Concerning to the interaction mechanisms with the host, the virus binds to target with its Spike proteins on its surface and uses it as an anchor. The Spike protein targets the ACE2 cell receptor and enters into the cells by using a special enzyme (TMPRSS2). Once the virion is quietly accommodated, it releases its RNA. Proteins and RNA are used in the Golgi apparatus to produce more viruses that are released. Concerning the therapies, different protocols have been developed in observance of the disease severity and comorbidity with a consistent reduction in the mortality rate. Currently, different vaccines are currently in phase IV but a remarkable difference in efficiency has been detected concerning the more recent SARS-CoV-2 variants. Conclusions: Among the many questions in this pandemic state, the one that recurs most is knowing why some people become more seriously ill than others who instead contract the infection as if it was a trivial flu. More studies are necessary to investigate the efficiency of the treatment protocols and vaccines for the more recent detected SARS-CoV-2 variant.

## 1. Introduction

**Figure 1:**
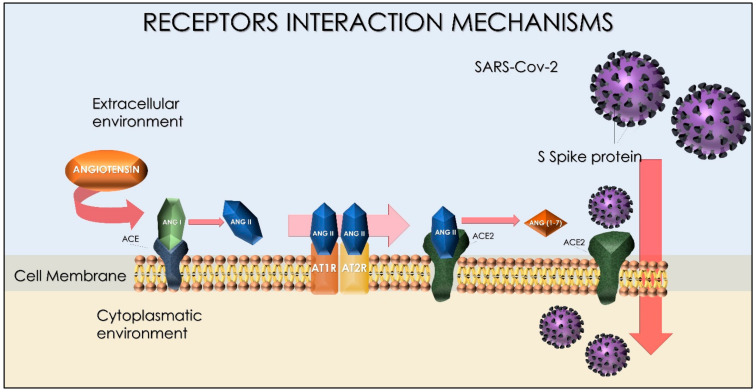
Synthesis of the ACE2 receptors interaction mechanisms of the SARS-CoV-2 viral vector.

Since December 2019, severe acute respiratory syndrome coronavirus 2 (SARS-CoV-2) has spread rapidly from Hubei province in China all over the world [1]. The COVID-19 disease is an acute respiratory infectious disease that is mainly transmitted through the respiratory tract more commonly by the release of droplets and direct/indirect contact through the respiratory tract and salivary secretions. The virus’ transmission via aerosols and airborne is suspected to be a key component for transmission [2,3]. In this manner, the droplet’s size, the settling speed, and the moisture content of the air are able to determine diffusion and suspension capabilities in the environment. In the lowest percentage, exposure occurred via the fecal–oral way [4]. The SARS-CoV-2 is able to complete the prodromal phase approximately after 2–14 days from exposure, with a wide variability according to the patient’s age and comorbidities. Similarly, to the MERS-CoV, the SARS-CoV-2 has a tropism towards the airways and exhibits a wide range of symptoms from mild respiratory infections to severe acute respiratory syndrome. The most common presentation is characterized by fever, dry cough, fatigue, and wheezing, and pneumonia as severe symptom [5]. The patient’s isolation is indicated to reduce the transmission to other subjects, such as healthcare workers [6,7,8]. Due to the pandemic, isolation restrictions have been adopted for symptomatic and asymptomatic patients in order to reduce vector diffusions to the general population [9,10,11,12]. In terms of charges for the healthcare system, the infectious diseases produces an intense economic and management load, as well as for the seasonal influenza with huge economic [13] and social burden [14]. The widespread preventive vaccination is able to reduce healthcare costs and plays a vital role in protecting fragile subjects from viral infections efficiently and sustainably, with a consistent reduction in the transmission’s impact within the population [15]. The SARS-CoV-2 is characterized by four structural proteins: Spike protein (S), which play a key function for the virus penetration in the host cells; envelope protein (E) and membrane protein (M) that are deputed to the virus infectivity, with a core formed by nine accessory nucleocapsid protein (N) [16,17,18]. The S protein is a transmembrane glycoprotein modified by many glycosylation processes [19]. Currently, the widest parts of SARS-CoV-2 vaccines are directed against the Spike protein, while the virus’s penetration is performed by the fusion of two different subunits [20]. For this scope, a key role is played by the neutralizing antibodies of anti-SARS-CoV-2, a particular type of IgG-ABs able to inactivate viral infections of the target cells [21]. SARS-CoV-2 neutralizing antibodies mainly interfere with S proteins present on the viral membrane in two ways: (1) The prevent the binding of the S1 subunit to the ACE2 receptor present on cells target; (2) they block the change in conformation of the S2 subunit and, thus, prevent the virus from entering the target cell [22] (Figure 1).

## 2. COVID-19 Variants and Antibodies’ Response

According to WHO guidelines, a total of three families of variants have been considered: variants of concern (VOC), variants of interest (VOI), and variant under monitoring (VUM) [23].

### 2.1. Variants of Concern (VOC)

SARS-CoV-2 variants have been classified by following changes: (1) dangerous changes in epidemiology or enhanced contagiousness; (2) changes in clinical disease’s features or increased virulence; (3) reduction in the efficacy of therapies, vaccines, and diagnostics or social and public health interventions. Currently, the more relevant VOCs are Alpha, Beta, Gamma, Delta, and Omicron [23] (Table 1). The Delta variant (AY.1 or B.1.617.2.1) and Delta Plus is increasing its spread. This variant is characterized by two mutations on the S protein that were present in previous COVID-19 forms. Currently, this variant represents the 6% of the COVID-19 cases in UK but it is too far from other variants in term of global circulation. In US, almost all COVID-19 cases involve the Delta variant but “Delta Plus” is occasionally registered and it is not considered a VOC or VOI by the WHO. The Delta variant is prevailing and continuing its evolution, and the WHO keeps it under investigation [24]. The WHO on 26 November 2021 stated that variant B.1.1.529/Omicron became a concern [25] while a total of five main subvariants have been detected. The omicron variant is characterized by other S protein mutations that determine virulence increases and disease’s gravity. The Omicron variant is associated with a 2.4-higher reinfection rate compared to the previous variants. This aspect is mostly induced by the high mutation multiplicity of the omicron variant. Currently, new findings on antibodies protection offered by vaccines for the omicron variant are emerging in the literature [26] in relation to Omicron’s spread becoming a concern to scientists from worldwide [27].

### 2.2. Variants of Interest (VOI)

The variants of interest (VOI) represent variants with genetic mutations that cause changes in transmissibility, disease’ severity, evading diagnostics, therapeutic solutions, and immune defenses. Moreover, they have been recognized in multiple countries as a cause of transmission or COVID-19 clusters correlated with an increasing number of cases over time with dangerous epidemiological repercussions for public health: ETA, IOTA, KAPPA, LAMBA, and MU [23] (Table 2).

### 2.3. Variants under Monitoring (VUM, Formerly Called “Alerts for Further Monitoring”)

A SARS-CoV-2 variant (Table 3) shows genetic mutations that interact with the virus’s characteristics resulting in future risks, without an obvious epidemiological repercussion; therefore, new tests will be necessary to document the pandemic’s evolution [23].

The COVID-19 variants’ spread such as Alpha (English variant), Beta variant (South Africa variant), Gamma variant (Brazilian variant), and Delta (India variant) deserves attention since the mutations cause more virulence, contagiousness and antibody resistance [28]. The Alpha variant (B.1.1.7.) appeared first in December 2020 and is approximately 82% faster than the original virus and Beta or B1351 in January 2021. The Alpha variant is 50% faster than the original strain, while the Gamma or P.1 showed an increase in virulence of 161%. Eepsilon presents two mutations, B 1429 and B 1427, in the USA since March 2020 and showed an increase in virulence of 20%. Delta B 1617.2 originated in May 2021 and reported an increase in virulence of 198%, which is quicker than the original strain [29,30,31,32,33,34,35]. The Spike protein (S) gene’s first mutation was positioned at codon-614 (D614Gis), and it is correlated with a substitution of aspartic acid (D) with Glycine (G) causing an expansion of the S receptor-binding region (RBD), enabling a more efficient and tighter binding of the virus with the ACE2 receptor [36]. This novel G mutation has spread to 100% of total strain in USA, Europe and in many countries, even if it was rare in Wuhan [36]. This strain appeared in Vietnam very early during March 2020 and was transmitted by travelers from Europe [37,38]. According to the WHO, in June 2021, there were a total of seven variants: Epsilon (B.1.427 e B.1.429); Eta (B.1.525); Iota (B.1.526); Kappa (B.1.617.1); Zeta (P.2); Theta (P.3); e Lambda (C.37) [39]. The strains Kappa and Eta showed a D614G mutation [37,38], which also produces alterations of other codons that change Spike protein receptor-binding regions, improving virulence performances, infectiveness (N501Y and E484K) or virus resistance to vaccines and serotherapy antibodies (E484K, K417N/T, and L452R) [38]. In particular, the E484K mutation makes the virus more resistant to the neutralizing antibodies of existing vaccines and therapies [40,41,42,43]. Sequencing the virus’ genome, many other mutations on S and N genes and other genes were reported in the literature. Currently, real-time PCR (Polimerase Chain Reaction) facilities represent a very useful instrument in contrast to the pandemic emergency by detecting variants and their level of danger in samples of infected cases [44]. The WHO recommends either NGS (Next-Generation Sequencing) or the Sanger method as procedures and analyses for detecting variants of SARS-CoV-2 [44]. Moreover, the real-time PCR technique remains the most appropriate procedure for monitoring viral mutations. Currently, several studies [45,46,47,48,49,50] as well as commercial kits [51,52,53,54] for real-time PCR techniques are now available for identifying mutations and other SARS-CoV-2 variants. This methodology allowed clinicians and health-care providers to discern the SARS-CoV-2 from SARS-CoV-1, MERS, and common influenza [55]. The Lambda variant (C.37—code GISAID GR/452Q.V1) requires more attention, which was first discovered in Perù in December 2020, reaching 82% of cases in May–June 2020; WHO classified Lambda as VOI in June 2021. Lambda may also be more threatening than Delta, which is 60% more contagious. Present in 30 countries according to the international database GISAID, most Lambda cases spread to South America (about 4000), peaking on 3 May 2021 and then tapering off slowly and then collapsing from 19 July 2021; it continually decreased and no changes have been registered from March [56]. The Lambda variant is characterized by three Spike protein mutations (RSYLTPGD246-253N, L452Q, and F490S). This major infectivity is due to two mutations in the Spike’s receptor binding, which are T76I and L452Q; the last one is similar to the Delta variant mutation L452R and it could explain the increased contagiousness of both Delta and Lamba variants. The resistance to antibodies is caused by the RSYLTPGD246-253N mutation (seven amino-acid deletions at the region of the N-terminal domain of the Lambda Spike protein). Additional genetic changes are T76I, G75V, F490S, del247/253, D614G, and T859N [57]. The Lambda variant may decrease about 4.6-fold the efficacy of neutralizing antibodies produced by mRNA vaccines. To a greater extent than the Beta variant, this one is known for its mutagenic capacity, making it resistant to immune defenses [57]. All vaccines developed effective neutralizing antibodies [57,58]. According to a new study on individuals that were just infected, the largest difference between BNT162b2 and ChAdOx1 nCoV-19 vaccines against Delta and Alpha variants happens after the first administration while a few differences have been registered after two doses of vaccine; in particular, both vaccines are efficacious against variants with a lower response for the Delta variant. Hence, this study proposes double-dose administration for the most vulnerable people. A study found that COVID-19 hospitalizations and deaths (more than 9/10) occurred in unvaccinated or not fully vaccinated people in 50 states of USA until July 2021 [59].

The current licensed vaccines seems to reduce severe COVID-19 complications and the mortality rate of SARS-CoV-2, with both Delta and other variants [60]. Being more contagious, the Delta variant could increase infection possibilities among completely vaccinated people, transmitting it more efficiently to others [61]. According to a study conducted on health care professionals at a tertiary care center, a significant higher humoral immunogenicity was registered in the SARS-CoV-2 mRNA-1273 vaccine compared with the BNT162b2 vaccine at all ages and in both infected and uninfected individuals [62]. In addition, antibody amounts in formerly non-infected participants were more elevated among in the people under 35 years old, supposing that the increase in age negatively influences the antibody’s response [62]. A reason for this difference could be correlated to a longer period between priming and boosting for mRNA-12733 and the highest mRNA level in Moderna vaccine when likened to Pfizer-BioNTech [62]. The Lambda variant was declared more infectious by WHO even if its aggressiveness was not demonstrated [63]. At the current state, the part played by children in the virus’s spread has not been clarified [64]. In recent results, the vaccines against COVID-19 are well tolerated by subjects between twelve and sixteen years old [65,66,67].

## 3. Mechanisms of Relationships between ABO Blood Groups and COVID-19

During SARS-CoV1, the association between COVID-19 infection and blood groups has been investigated in several studies, and many of these suggest the existence of an increased susceptibility to infection in blood group patients 0. This possible association has also been studied for SARS-CoV-2 [68]. There are different hypotheses about the mechanism and interaction between ABO blood type and SARS-CoV-2. Anti-A and/or anti-B antibodies expressed in group 0 might bind to A and/or B antigens expressed on the viral envelope, promoting viral neutralization [69].

There is emerging evidence that ACE-2 receptor-binding proteins of SARS-CoV-2 share a similarity with an ancient lectin family known to bind blood groups. The possible correlation between blood group A and SARS-CoV-2 could be the link between SARS-CoV-2 RBD and blood group A expressed on respiratory epithelial cells [70]. Moreover, this condition could be correlated to a greater angiotensin enzyme function, VWF, factor VIII, and the severity of the clinical presentation of COVID-19 disease in group A. In fact, more cardiovascular complications occur in these subjects. There is no confirmed evidence but the ABH glycans present in plasma, secretions, and on cell surfaces could play a role in COVID-19 disease severity. In this manner, the identification of the Lewis blood group and the “secretor phenotype” of ABH glycans level could play a key role for the severe development of the disease [71,72].

## 4. Spike Protein Interactions

**Figure 2:**
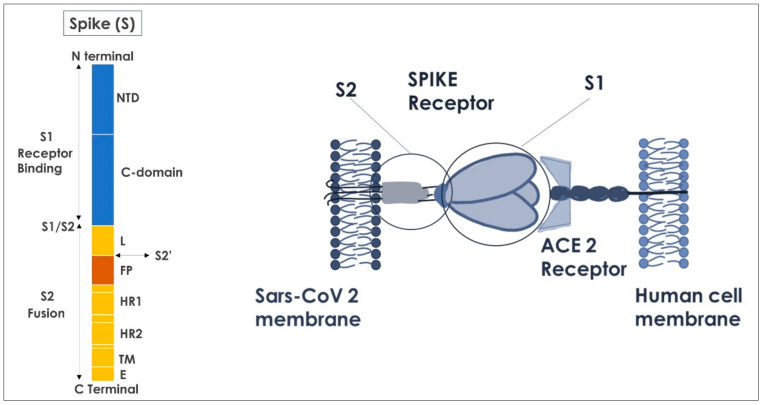
Schematic diagram of the structure of SARS-CoV-2 and its Spike protein. The protein S is placed inward on the envelope to form a crown structure consisting of two subunits, S1 and S2. The protein hemagglutinin esterase (HE) is only present in A-series betacoronaviruses. Figure designed by Giovanna Dipalma [45].

The definition of coronavirus is correlated to the presence of 20–40 nm-long Spike glycoprotein, which protrude from the viral envelope (Figure 2) [73].

**Figure 3:**
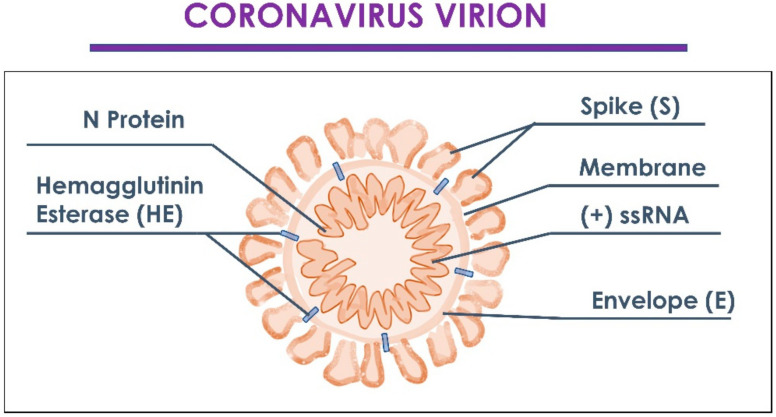
SARS-CoV-2. Spike. RNA and N Protein, Envelope, Protein M, Hemagglutinin-esterase dimer (HE). Figure designed by Giovanna Dipalma [76].

The SARS-CoV-2 genome encodes four structural proteins: Spike, envelope, membrane, and nucleocapsid and 16 non-structural proteins [74]. Spike proteins are formed by an extracellular N-terminus, a transmembrane ™ domain, and an intracellular C-terminal segment. Moreover, the Spike protein weight is of 180–200 kDa (Figure 3) [75].

**Figure 4:**
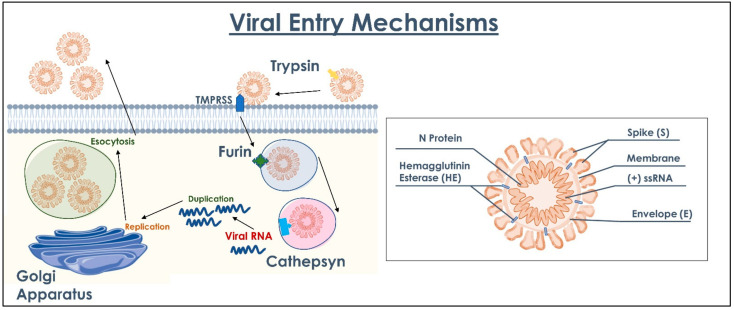
Activation scheme of Spike protein. Protein S is placed outward on the envelope to form a corona structure. Protein S consists of two S2 subunits fused. Activation of trypsin, furin, TMPRSS, and cathepsin in fused host cells. Characters designed by Giovanna Dipalma.

The extracellular domain is glycosylated (Figure 2) with N-linked glycans. Cysteine residues in the cytosolic portion are palmitoylated [77,78]. The Spike protein (Figure 3) ectodomain is heavily glycosylated with heterogeneous N-linked glycans and exists in a prefusion form and a post-fusion form. The oligosaccharides could influence priming by host proteases and determine antibody recognitions. The Spikes share 93% and 97% of amino-acid genome with the genome of Pangolin CoV and BatCoV RaTG13 and Pangolin-CoV [79,80,81,82]. Mutations present at the interface between the Spike protein and the ACE-2 receptor may affect vaccine performance and drug design at the protein–protein interaction interface [83]. During the virus’ transmission, the S2 subunit is released after the cleavage of Spike proteins into S1 and S2 subunits. The S1 subunit contains two domains, termed the N-terminal domain (NTD) and the C-terminal domain (CTD), and these are located in the receptor-binding domain (Figure 4) [84,85,86,87]. In particular, to enter into host cells, SARS-CoV-2 firstly needs the binding of the Spike protein to a cell surface’s receptor, namely ACE2 mediated by the RBD of S1. The RBD at the apex of S1 undergoes a hinge-like conformational movement that momentarily exposes (open state, “up”) or hides (closed state, “down”) subdomains required for receptor binding, whereby the open state allows receptor engagements [88]. The ligation of the S1 RBD to the ACE2 enzyme exposes a cleavage site on S2 that is acted upon by host-cell proteases such as TMPSSR2 to initiate the cell-entry process [89]. The ammino-acidic sequence is 72% similar to the RBD sequences of SARS-CoV and SARS-CoV-2, with highly similar 3D structures.

Homology models and biophysical properties suggest that the SARS-CoV-2 RBD domain binds ACE2 with a 10- to 20-fold-higher affinity than SARS-CoV [90]: SARS-CoV-2 has a distinct furin cleavage site (Arg-Arg-Ala-Arg) at residues 682–685, which increases the likelihood of cleavage by furin-like proteases, which also enhances its infectivity [91]. The furin cleavage site expands the versatility of SARS-CoV-2 in cellular protease cleavage, as well as the potential tropism and transmissibility due to the widespread expression of furin in cells. This means that newly synthesized virions can be secreted in a “preactivated” state, ready to fuse with and infect other cells without binding to cellular receptors such as ACE2 [92]. The S protein of SARS-CoV is cleaved by transmembrane protease/serine subfamily member 2 (TMPRSS2), which are most expressed on the epithelial cells of respiratory tracts [93]. After Spike cleavage, there are some important structural changes that allow the fusion of viral and host cell membrane with the entry of a viral core [94,95]. ACE2 is a dipeptidyl carboxypeptidase, mainly a type 1 TM protein of 805 amino acids. It exists in the form of a zinc-binding domain and is mainly expressed in the lung, heart, kidney, testis, and gastrointestinal tract [65]. ACE2 contains two viral hotspots of lysine residues that appear to be critical for CoV binding: Two ACE2 alleles, rs73635825 (S19P) and rs143936283 (E329G), may confer resistance to SARS-CoV-2 infection. [96]. ACE2 has been widely recognized as a receptor binded by Spike proteins [97]. In lung disease, the loss of ACE2 activates the renin–angiotensin system, enhances vascular permeability and pulmonary edema, and contributes to the pathogenesis of severe lung injury [98]. SARS-CoV-2 infection downregulates the ACE2 receptor, which favors the progression of thrombotic processes [99]. Viral prion-like domains (PrDs) have been identified in the ACE2 protein within the α1 helix (aa 40–65 and 93–106) [100]. PrDs have been proposed as novel regulators of virion assemblies, playing a role in virus–host cell interactions [100]. Overall, dementia and hypertension significantly upregulated ACE2 vasculature expression, suggesting that SARS-CoV-2 may be more likely to encounter its key cell-binding targets in individuals with these comorbidities. The SARS-CoV-2 Spike protein is responsible for clinically observed edema, whereas systemic diffuse hyperinflammation is caused by increased secretions of proinflammatory cytokines in the endothelium. Viral pathogens negatively impact the blood–brain barrier (BBB), either through direct interactions with the endothelium or stimulating host immune responses, increasing the expression of proinflammatory cytokines, chemotaxis cytokines, cell adhesion molecules, and ultimately leading to the loss of the structural and functional integrity of the BBB. The disruption of the BBB releases the free passage of viral fragments and infected immune cells into the brain parenchyma, further increasing levels of inflammatory mediators and exacerbating the disruption of endothelial barrier functions [58,101,102,103]. In individuals recovering from COVID-19, adaptive immunity to SARS-CoV-2 is primarily mediated by CD4+ cells with a repertoire of T cell receptors specific for the S epitope, resulting in robust neutralization IgG, IgM, and IgA antibodies producing trimers of the ectodomain of RBD and S1 [104,105] while individuals exhibit b-cell monoclonal antibody neutralizing activities, which bind ACE2 to RDB and the NTD of S, indicating that the two S epitopes are highly immunogenic at the apex of S [106,107]. Currently available tests and previous serological studies have dosed antibodies against the N protein, RBD, or S protein [104,108].

## 5. Serological IgM, IgG, and IgA

The levels of peripheral blood antibodies monitoring, i.e., immunoglobin (Ig) G, M, and A specific to SARS-CoV2, represent a different method for diagnosing SARS-CoV-2 infections, including asymptomatic carriers, and a simple method for controlling antibody responses in convalescent patients/vaccinated subjects [109,110]. In the literature, it was reported that an important antibody responses occur between 17 and 23 days after disease onset while they are stronger but slower in more severe patients [111]. Since IgM, IgG, and IGA are the principle antibodies involved in the SARS-CoV-2 infection, they are used to define and monitor the immune response of patients [109,112]. After three days from the onset of symptoms or one week after contracting the infection, IgM antibodies are generated, and after reaching their peak, the production of IgG follows. Specific IgMs involve early antibody responses that start and peak within 7–12 days and decrease meaningfully after 18 days; by contrast, specific IgG antibodies develop a few days later (ranging from 10 to 18 days) and do not decrease, and they persist throughout a lifetime as protective antibodies [113]. The tracking of high and persistent levels of antibodies against SARS-CoV-2 and, in particular, of anti RDB-IgG neutralizing antibodies, which recognizes the Spike protein of SARS-CoV-2 and prevents cell infections, representing a strong indication that an immunized host could resist SARS-CoV-2 infections. Neutralizing antibodies block a pathogen from infecting the host by inhibiting the molecules on the pathogen’s surface used to enter the cells [114]. Therefore, the neutralizing antibodies result in lifelong immunity with respect to SARS-CoV-2 infections. The patients with a high level of IgG are able to neutralize the virus efficiently; thus, monitoring these antibodies is both a method of detecting previous infections or immunizations and an immunity passport. Anti–SARS-CoV-2 Spike protein IgG levels and neutralizing antibodies in the plasma of infected people are connected [115]. According to some studies, high levels of anti-RBD IgG have been found in COVID-19 subjects, but they do not neutralize the virus, such as anti-RBD IgA and IgM [116]. The IgAs represent the main immunoglobulins involved in the digestive and respiratory immune system, so they should be considered in serological tests along with IgG and IgM [117]. The stimulation of mucosal immunity via IgA may be relevant in preventing SARSCoV-2 infections [118]. Indeed, the virus recognizes and infects respiratory epithelial cells by binding to the angiotensin-converting enzyme-2 (ACE2) protein on the surface of type-2 alveolar cells [119]. Furthermore, besides typical respiratory symptoms, digestive symptoms including nausea, vomiting, diarrhea, and anorexia may occur in COVID-19 patients. Some patients may develop digestive symptoms in the absence of any respiratory symptoms [120,121]. Therefore, IgA assays could be useful, along with IgG and IgM, for monitoring and recognizing patients with atypical symptoms and in paucisymptomatic cases (including mild conjunctivitis, low fever, and digestive symptoms) or in suspected subjects with a negative RT-PCR result for a naso-pharyngeal swab [122,123]. To this end, anti-SARS-CoV-2 humoral responses can support and enhance COVID-19 diagnoses, including subclinical and asymptomatic cases. The production of IgA is peculiar and, initially, parallel IgM kinetics with IgA and IgM levels increased since days 6–8 from symptom onset, and IgA showed persistently higher levels over 38 days, with a peak level at days 20–22, whereas IgM levels peaked at days 10–12 and decreased mostly at day 18 [124,125]. Overall, the comprehension of SARS-CoV-2 diagnostic tests is still evolving and a clear knowledge of Ig kinetics will be useful for the interpretation of tests and of serologic results [113]. Indeed, although the timing of Ig production (from 4 days after symptoms onset to 10–14 days) limits its applicability for COVID-19 diagnosis in the acute phase [28,126], the detection of anti-SARS-CoV-2 IgM and/or IgA antibodies may represent an important tool for recognizing patients with the RT-PCR negative gap. Hence, the combination of serological tests with molecular tests will improve the diagnosis of COVID-19 patients, and as main indicators of immune development, it will be useful for controlling the pandemic [113]. The specimens for SARS-CoV-2 tests are acquired from the upper respiratory tract, nasal aspirate and wash, nasopharyngeal/oropharyngeal buffers, saliva, or lower respiratory tract. Additionally, specimens from the lower respiratory tract are suggested to decrease false negatives [127]. To this end, three different approaches are adopted: (1) RNA detection test through nucleic acid amplifications using the RT-PCR procedure, (2) antigen tests based on the recognition of a specific surface protein viral antigen, and (3) antibody tests based on the recognition of specific antibodies against SARS-CoV-2. Salivary oral-pharyngeal buffers and serological tests are used for diagnoses; IgM and IgG are measured by using nucleocapsid protein (NP) cross-reactive of another SARSr-CoV Rp3, which is similar for 92% relative to the 2019-nCoV [127,128,129].

The ELISA test can identify IgM and IgG simultaneously with the antibody reaction against rNPs purified from SARSCoV-2, i.e., the N helical capsid proteins. In this sense, the PCR is more sensitive compared to the ELISA test for IgM five and a half days before the onset of symptoms [130]. Furthermore, immunosorbent assay (ELISA) analyses for Spike protein S and N protein has high specificity (99%) for the antigen’s detection, providing final SARS-CoV-2 diagnoses [131]. Immunodominant antigens may be present at the beginning of the infection because a major antibody response against N capsid protein has been detected by other assays. Hence, some rapid antibody IgM and IgG tests were produced during the SARS-CoV-2 infection in order to make an early diagnosis of COVID-19 [132]. The sensitivity was better with a combined IgM-IgG antibody test than with tests performed individually for detecting IgM or IgG [133]. In particular, the main differences between the antigenic and molecular tests are as follows:

- The antigenic test is performed by a swab on nasal mucosa or oropharyngeal region by identifying SARS-CoV2-specific Spike glycoproteins (S). Nevertheless, this technique may provide false negatives. In many cases, the test should be subjected to a repetition in the following days [130].
- The molecular test, instead, identifies SARS-CoV-2 genomes in the substance and are present in swabs [130].

SARS-CoV2 infection could produce false negative results in tests, especially in case of nasopharyngeal swabs performed too early concerning the prodromal phase [133,134,135,136]. Repeated swabbing and the consideration of a deeper respiratory tract sample are recommended [111]. As prevention is the first resource, testing criteria are being developed and progressed [134].

Children can be infected with SARS-CoV-2, but most of the times, they are asymptomatic or have minor symptoms; in fact, infants under 12 months of age do not develop serious pathology. As with adults, a SARS-CoV-2 infection in children is diagnosed by nucleic acid amplification test (NAAT) using reverse transcriptase viral polymerase chain reaction (RT-PCR) by taking a sample from the upper airways [135].

## 6. Anti-Spike and Anti-N Difference

Currently, immunoassays have become fundamental tools for detecting SARS-CoV-2 infection and to survey its spread. The antigen test detects SARS-CoV-2 antigens, while serology tests detect anti-SARS-CoV-2 antibodies fighting against SARS-CoV-2 [137]. The main virus’s proteins considered as targets for antibody detection are the nucleocapsid (N) or Spike (S), thanks to the abundance of the former and the specificity of the latter [130,138]. Both proteins can be used in their full length or in a truncated version for antibody detection. Thus, immunoassays can detect either the viral structural proteins or seroconverted IgM and IgG antibodies, which can be found in the blood or the serum [139]. Several studies showed the presence in serum of IgG against N protein after 4 days since the outcome of SARS-CoV-2 disease and its seroconvertion on the 14th day [140]. However, studies using an antigen S are more sensitive than tests based on antigen N, with IgG tests performing better than IgM tests [141,142]. Nevertheless, all these serological tests can be used for the identification of SARS-CoV-2 infections [143]. An association between increased IgG versus S and a decrease in C-reactive protein was found. In fact, S-specific antibodies block entry into the target cell by inhibiting the binding of S proteins to the hACE2 cell receptor. Thus, S-IgG monitoring can be used as an aid in predicting prognosis. In contrast, a continued increase in IgG-N correlates with severe disease progression. This suggests that N-specific antibodies are probably not capable of interfering with virus entry into the cells [141,143]. Werner et al., emphasized the advantages of the simultaneous detection of Spike and nucleocapsid proteins in terms of increasing the detection sensitivity and testing accuracy. Moreover, this combination could be a method for reducing false-positive testing results [142]

The most frequent immunoassays used to recent COVID-19 diagnosis are chemiluminescence immunoassay (CLIAs) [138], ELISAs, rapid diagnostic tests, and the lateral flow immunoassays (LFIAs) [130]. Kyosei at al. recently developed a diagnostic ultrasensitive method by combining a sandwich ELISA and the thionicotinamide-adenine dinucleotide (thio-NAD) cycling reaction to quantify Spike S1 proteins [144].

## 7. COVID-19 Syndrome: Diagnosis and Treatment Procedure at Home and in Emergency

The role of humoral responses in COVID-19 has gained particular attention since the virus and virus-infected cells may strike the B lymphocyte production centers, causing a decreased number of specific antibodies against SARS-CoV-2. The active presence of neutralizing antibodies revealed to be a good resource to control SARS-CoV-2 infection; however, not all post-COVID-19 showed a sufficient number of neutralizing antibodies that can be used as anti-COVID-19 therapies [145,146]. The notorious COVID-19 cytokine storm is the main cause of the alveolar gas exchange mechanism. The scenario is characterized by the presence of uncontrolled high levels of pro-inflammatory cytokines and interleukins, such as as IL-6, interferon gamma (IFNγ), tumor necrosis factor alpha (TNFα) mainly produced by macrophage type 1, Th1 cells, and neutrophils. The highly affected microenvironment is then characterized by the accumulation of lymphocytic granulomatous, a viscous neutrophilic extracellular trap (NET) that induces massive thromboembolic events often associated with both reduced gas exchanges (O_2_/CO_2_) and alkalosis, which became hallmarks of a silent progressive organ decay known as “Happy hypoxia”. In this perspective, COVID-19 treatments might contemplate the possibility of enhancing an immune modulatory answer by stimulating the activity of immune-modulators cells such as B-lymphocytes or M2 macrophages [147,148,149]. According to the findings, SARS-CoV-2 is extremely adaptable and uses the angiotensin-converting enzyme 2 (ACE2) receptor as the main entrance into the cells and system. However, the virus is also facilitated by the presence of multiple genetic mutation on gene-regulating immunity and metabolic activities such as the those involved in homocysteine metabolism and blood coagulation. For instance, people with ACE2 polymorphisms who have type 2 transmembrane serine proteases (TMPRSS2) are at high risk of SARS-CoV-2 infections. Similarly, individuals with IL-6 polymorphisms (IL-6 -572 G-C rs1800796 and IL-6 -174 G-C rs1800795) are the most affected by the uncontrolled “cytokine storm” due to the hyper response of IL6 aggression, as well as those with mutations on IL 10, IL1b RN, and IL6R, which are genes in charge of immune modulation responses and are at great risks of severe collateral effects. Patients possessing a genetic make-up with specific mutated genotypes, either homozygous or heterozygous, may become an easy target for the COVID-19 infection [150,151,152]. The specific mutations evaluated on IL1b RN and IL6R indicate damages in the binding ability of IL6, IL1b, IL6R, and IL1RN. The Ingenuity Pathway Analysis (IPA) demonstrated that these mutations were implicated with SARS-CoV-2-infiltrating abilities as well as with neurodegenerative diseases mediated by neuroinflammation processes. Thus, the possibility of screening and confirming the presence of those mutations could be used as ready-to-go biomarkers for COVID-19 to respond, in a prompt manner, and to prevent multiorgan failure and death and commence the most suitable treatment strategies to improve patient prognoses. This allows reaching different clinical needs and realizing personalized medicine protocols [12,54] for chronic, age-related emergencies [153,154]. The COVID-19 infection is characterized by different syndromic pictures and of different grades of severity in which the following are distinguished (Table 4):

1. The less severe clinical form, which includes the paucisymptomatic condition characterized by anosmia and ageusia; the oligosymptomatic form, which includes minor respiratory problems, nasal congestion, conjunctivitis, pharyngodynia, cough, and gastrointestinal problems (abdominal aches, vomiting, and diarrhea), and neurological symptoms such as dizziness, syncope, and headache [155];
2. Clinical variants characterized by greater concerns and different stages. At the beginning, there are rapidly evolving pictures of the minor forms, described as follows: interstitial alveolar pneumonia with acute hypoxemic-hypercapnic pneumonia that may evolve to the most serious forms including sepsis, hypovolemic shock, and eventually multi-organ appearance syndrome (MODS).
3. High risk clinical pictures are essential criteria for emergency clinical diagnosis, and they are as follows: alteration of the sensory, tachypnea HR > 30 beats/min, dyspnea, dehydration, oligo-anuria, very high fever (>39 °C) and protracted > 7 days, persistent dry cough, hoarse voice, anosmia/ageusia, intense, exhaustion, extreme fatigue, and body aches [156,157,158].

**Table 4:** Summary of the COVID-19 treatment protocols.

COVID-19 SYNDROME TREATMENT	
RISK FACTORS hypertension heart disease diabetes mellitus obesity chronic lung disease chronic renal failure neoplasms smoke age > 50 years	CLINICAL SEVERITY LEVELS OF ARDS Mild ARDS—P/F between 200 and 300 mmHg with CPAP or PEEP > 5 cm H_2_O Moderate ARDS—P/F between 200 and 100 mmHg with CPAP or PEEP> 5 cm H_2_O Severe ARDS—including P/F < 100 mmHg with CPAP or PEEP > 5 cm H_2_O
LABORATORY RESULTS SpO_2_ < 90% PaO_2_ < 60 mmHg PaCO_2_ < 35 mmHg or >55 mmHg PaO_2_/FiO_2_ < 200 mmHg with CPAP or PEEP > 5 cmH_2_O Lactic acid > 3 mmol/L, high LDH Neutrophilia Severe lymphocytopenia Thrombocytopenia High level D- Dimers High ESR, High C reactive protein, low e-GFR, high fibrinogen, high CPK, high BNP High IL-6 Low Vitamin D3 *Instrumental tests* Positive CT thoracic with ground glass opacity indicative for interstitial bilateral alveolar pneumonia to be performed immediately in patients with P/F < 300 mmHg [159,160,161].	EMERGENCY THERAPY *Respiratory stabilization* On the territory SpO_2_ > 90 mmHg O_2_ with nasal cannula/face mask with reservoir/ventimask SpO_2_ < 90 mmHg CPAP 6–10 cmH_2_O/initial FiO_2_ 50–60% *In the Emergency Unit/hospital critical area* P/F > 350 O_2_ with nasal goggles/face mask with reservoir/ventimask P/F < 350 O_2_ at high flows/CPAP 6–10 cm H_2_O/initial FiO_2_ 50–60% P/F < 200 assess the need for early intubation P/F < 100 mandatory intubation with pronation cycles
PHARMACOLOGICAL THERAPY dexamethasone fl IV 4 mg: 1 fl IV morning and evening in 100 mL of saline pantoprazole 40 mg fl iv: 1 fl IV morning and evening in 100 mL of saline enoxaparin sodium fl sc: 1 mg/kg ceftriaxone fl 1 g IV: 2 fl IV acetylcysteine 300 mg fl: 1 fl IV morning and evening in 100 mL of saline [160,162]	HOME THERAPY Low molecular weight heparin enoxaparin sodium fl sc: 1 mg/Kg Cortisone methylprednisolone 16 mg tablet: 1 tablet morning and evening NSAID ketoprofen 200 mg cp: 1 after the main meal Antileukotrienic montelukast 10 mg cp: 1 cp 2 h after dinner Antihistamine ebastine 10 mg cp: 1 in the evening after dinner Antibiotic azithromycin 500 mg in the morning + levofloxacin 500 mg in the evening Antioxidant acetylcysteine 600 mg: 1 effervescent tablet every 12 h Antioxidant melatonin 2 mg tablet: 1 tablet after dinner Immunomodulator vitamin D3: 10,000 IU per day orally Antipyretic paracetamol 1000 mg cp: 1 cp la of if fever> 38.7, possibly repeatable within 24 h as needed Gastroprotector pantoprazole 40 mg cp: 1 tablet in the morning and in the evening [163,164]

The clinical severity grading of ARDS is as follows:

1. ARDS mild P/F between 200 and 300 mmHg with CPAP/PEEP > 5 cm H_2_O;
2. ARDS moderate P/F between 200 and 100 mmHg with CPAP/PEEP > 5 cm H_2_O;
3. ARDS severe P/F between <100 mmHg and CPAP/PEEP > 5 cm H_2_O [66,165].

## 8. Key Points in COVID-19 Patient Management

Patients with minor acute respiratory syndrome must be treated at home as if they were suffering from COVID-19 until proven otherwise by a molecular test (PCR), with therapy aimed at immediately counteracting any amplifications of the systemic inflammatory response (SIRS) and with an examination of the molecular swab for positivity. The patient with clinically involved COVID-19 is often characterized by a significant discrepancy between the clinical conditions, which seem reassuring, and the level of the objective severity of the disease with silent hypoxia, as detectable by objective laboratory-instrumental evaluations. It is necessary to promote every institutional awareness-raising action so that every patient with an acute respiratory syndrome from COVID-19 (presumed to be at home) is equipped with an oximeter and an oxygen cylinder with which the citizen-user reports CO118 SpO_2_ < 92% in ambient air, especially with a downward trend compared to the immediately preceding measurements, and a rescue intervention must be activated. The clinical emergency management of the patient with major acute respiratory symptoms and/or sudden and significant desaturation (SpO_2_ < 92%) should optimally identify the extent of acute respiratory failures by performing the EGA at the time of taking charge of the patient, and it should be possible to obtain the P/F (PaO_2_/FiO_2_) parameters as indicators of the effectiveness of pulmonary gas exchange at the capillary alveolus level, which allows setting the most appropriate timely therapy option. Acute respiratory distress syndrome (ARDS) and, in the most severe cases, multi-organ failure syndrome (MOFS) represent the main vital organ dysfunctional complication during severe clinical COVID-19. It can arise rapidly and immediately after the onset of dyspnea at an average of 8 days after the onset of the first symptoms [66,67,87,165].

## 9. Major Complications of COVID-19

The most common major complications are thromboembolic and massive pulmonary embolism [166,167], diffuse microvascular thrombosis [168], neurological complications resulting in strokes, encephalopathy, mono-polyneuritis, and pseudo-depressive forms that can arise from scratch or emerge from a pre-existing framework. The respiratory and hemodynamic stabilizations of the patient must be carried out with absolute timeliness regardless of the swab examination. In this manner, clinical conditions and precautions are as follows:

- Patient with P/F < 350 mmHg with CPAP or PEEP > 5 cm H_2_O should undergo NIMV;
- Patient with P/F < 200 mmHg with CPAP or PEEP > 5 cm H_2_O should be evaluated for early intubation;
- Patient with P/F < 100 mmHg with CPAP or PEEP > 5 cm H_2_O must be immediately intubated and subjected to cycles of pronation;
- The patient with COVID-19 should receive low molecular-weight heparin therapy early via the sc route, based on body weight [66,67,87,165].

## 10. Anti-SARS-CoV-2 Vaccines

The current literature demonstrated that vaccines are effective because they expose the host subject to pathogenic antigens and activate the immune system and immunological memory [169]. The immune system is stimulated to produce serum IgG [170].

The two vaccines’ antibody responses have been analyzed and Comirnaty by BioNtech and Pfizer received the AIFA’s approval on 22 December 2020 and Moderna was authorized on 7 January 2021. Both vaccines were approved by the Food and Drug Administration (FDA) and the European Medicines Agency (EMA) [171]. They are based on messenger RNA (mRNA) molecules that encode a protein present on SARS-CoV-2. The two vaccines are administered in two doses at least 21 days apart for the Comirnaty vaccine and at distance of 28 days for the Moderna vaccine [172]. Following contact with a microorganism, the immune system develops antibodies, which tend to decrease progressively. Several studies have stated that immunity against SARS-CoV-2 persists for several months [173,174,175]. Vaccines have increased life expectancy over the last 100 years because they have allowed the elimination of many infectious diseases [176]. Vaccines include viral vectors, inactive viruses, subunit vaccines, but with the development of nanotechnology, vaccine development is much faster [177]. The vaccination of 70% of the population allows us to obtain herd immunity [178]. On 12 November 2020, the World Health Organization drew up a list of vaccines in which 48 were undergoing clinical evaluation and 11 are currently in phase III studies [179].

### 10.1. SARS-CoV-2 Vaccine Candidate Phase III Clinical Trials

#### 10.1.1. Vaccine BBIBP-CorV; Developer: Beijing Institute of Biological Prodducts/Sinopharm

An isolated SARS-CoV-2 virus from a hospitalized COVID-19 patient and then inactivated with β-propionolactone was used to develop this vaccine [179]. Electron microscopy was used to assess the integrity of the virus [180]. In Abu Dhabi, 15,000 people are participating in a Phase III study, 5000 are receiving placebo, 5000 are receiving BBIBB-CorV, and the remaining participants are receiving another inactivated vaccine manufactured by Sinopharm [179].

#### 10.1.2. Vaccine Untitled; Developer: Wuhan Institute of Biological Products/Sinopharm

The WIV04 strain of the SARS-CoV-2 virus was isolated in a patient from the Jinyintan hospital in Wuhan, inactivated with β-propiolactone, and then ultracentrifuged [181]. Three international clinical trials on the vaccine are ongoing in Peru (NCT04612972), Abu Dhabi (ChiCTR2000034780), and Morocco (ChiCTR2000039000) where patients over 18 years of age have been enrolled [181].

#### 10.1.3. Vaccine: Ad5-nCoV; Developer: CanSino Biological Inc./Beijing Institute of Biotechnology

The vaccine consists of a non-replicating type 5 adenovirus (Ad5). This vaccine is in phase III clinical trials.

#### 10.1.4. Vaccine: CoronaVac (PiCoVacc); Developer: Sinovac

The CN2 strain of the SARS-CoV-2 virus was isolated to produce this vaccine and was stabilized and inactivated using β-propiolactone. The integrity of the inactivated virus was ascertained with electron cryomicroscopy [182].

Coronavac, manufactured by Sinovac, is given as a double dose two weeks apart. It contains 3 μg/0.5 mL of inactivated SARS-CoV-2 virus and aluminum hydroxide for adjuvants (Table 5) [183]. In this emergency situation, CoronaVac has been approved in China. Three Phase III clinical trials are being conducted in Brazil (13,000 health workers enrolled), Indonesia (1620 people enrolled), and Turkey (13,000 people enrolled) in people aged 18 to 59. From initial data, the vaccine prevents severe and minor illnesses and is approximately 50.4% effective [184]. Coronavac has an advantage that it can be stored in the refrigerator at 2–8 °C, making it easier to use. The study conducted in Brazil stated that the Cornavac vaccine is 78% effective in preventing mild forms of COVID-19 and 100% in moderate and severe forms [183], while the study conducted in Turkey found a 90% efficacy in preventing mild forms [183].

#### 10.1.5. Vaccine: Sputnik V; Developer: Gamaleya Research Institute

The Sputnik V vaccine features two non-replicating adenoviral vectors with two different adenoviral vectors (recombinant Ad26 and recombinant Ad5). The recombinant adenovirus Sputnik V (Ad) combined the Oxford–AstraZeneca chimpanzee adenovirus (ChAdOx), the Janssen vaccine (Ad26), and the CanSino vaccine using Ad5 (Table 6) [185]. Intermediate studies conducted by the Gamaleya National Center of Epidemiology and Microbiology in Moscow and the Russian Direct Investment Fund have shown that the vaccine has an efficacy of 91.4% [186]. It is not known if the vaccine is effective on the new strains; therefore, new studies are needed, although it presents a good chance as it is created with two viral strains. The vaccine should be stored at 2–8 °C for global distribution [187].

#### 10.1.6. Vaccine: NVX-CoV2373; Developer: Novavax

The Novavax vaccine consists of 5 μg of NVX-CoV2373 + 50 μg of adjuvant Matrix-M1 and is administered intramuscularly. Protein S was purified, leading to the formation of nanoparticles [188]. A study was conducted to evaluate the efficacy of two doses of this vaccine in patients infected with SARS-CoV-2. A Phase III clinical trial is underway in the United States and Mexico and 30,000 people have been enrolled. From the first set of data, the vaccine has been shown to be 90% effective and is effective against the variants currently in circulation [130]. In addition, the emergency use of this vaccine has been authorized in Indonesia, USA, Europe, and other countries [130].

### 10.2. SARS-CoV-2 Vaccines Phase IV Trials

The vaccines that have been approved in phase III and, therefore, can be distributed; to date, there are five, which are listed as follows (Table 7) [76]. Vaccine: BNT162b2; developer: Pfizer/BioNTech/Fosun [189]. Vaccine: Spikevax (previously COVID-19 Vaccine Moderna); developer: Moderna/NIAID [190]. Vaccine: Vaxzevria ChAdOx1-S; developer: Astrazeneca AB [191] and vaccine: Ad26.COV2-S; developer: Janssen-Cilag International NV [192]. The phase 4 study begins on 8 February 2021 and the estimated end date of the study is December 31 2024; the safety and efficacy of the vaccine against COVID-19 will be evaluated. Enrolled subjects are over 18 years of age and will be followed up 2 years after the administration of the first dose of vaccine. In addition, to assess safety, the subjects enrolled will undergo periodic visits for 3 months after the first vaccination [193]. Generally, the phase 4 trials evaluate the following [193]: the minimum level of antibodies needed to protect against infection (24 months after vaccination), the SARS-CoV-2 positive patients from the first vaccination up to 24 months, the local and systemic reactions to vaccination (from the first vaccine up to day 90), and the adverse events (from the first vaccine up to day 90).

#### 10.2.1. Vaccine: BNT162b2; Developer: Pfizer/BioNTech/Fosun

The vaccine consists of mRNA encapsulated with lipid nanoparticles. The first vaccine to be approved by the Italian Medicines Agency is Comirnaty, the use of which is recommended at two doses 21 days apart (Table 8). The first subjects to be vaccinated were health workers who joined the vaccination campaign, which began on 27 December 2020 [194,195]. The virus invades the host cell using the Spike protein, which is used to create the vaccine [196]. The vaccine consists of mRNA encapsulated with lipid nanoparticles [197]. The mRNA contained in the vaccine encodes the Spike protein [198]. Furthermore, the lipid nanoparticle that protects the mRNA from degradation was used to allow the mRNA to arrive in the host cell after intramuscular injections [199]. The mRNA is translated into the host cell producing the Spike protein and exposed on the membrane. The exposure of the protein on the membrane induces immune system activations and responses [200]. Studies in healthy adults vaccinated with two doses of BNT162b2 showed that the vaccine produced a high antibody response against SARS-CoV-2 [201,202]. The vaccine is 95% effective in the population over 16 years of age [201]. The Italian Medicines Agency has recommended the administration of two doses even if the data of clinical studies affirm that the subjects who have administered the vaccine have protections against COVID-19 even after a single dose [203,204]. Healthy children infected with SARS-CoV-2 have different symptoms from adult patients [205,206]. Children generally have mild symptoms, although the presence of pathologies such as neurological changes can cause serious complications [207,208]. Persons under the age of 16 have not been vaccinated as the SARS-CoV-2 vaccine was not authorized [201]. To date, there are data on the safety of vaccination for BNT162b2 (Pfizer-Bio-N-Tech) for healthy adolescents between the ages of 12 and 15. A study showed a high efficacy of this vaccine as the subjects enrolled were not infected with the virus after more than 7 days from the second administration of the vaccine [201]. Furthermore, safety has been demonstrated in subjects between 12 and 15 years of age [201]. Similarly to adult studies, common side effects were mild to moderate pain at the injection site (86%), fatigue (66%), headache (65%), and fever ≥38 °C (20%) [209]. This vaccine allows the generation of an immune response against the virus without infecting the organism [210]. The vaccine must be stored at −70 °C and this is a disadvantage of this vaccine; therefore, efforts are being made to keep it at 4 °C for five days [211]. Symptoms reported following vaccine administration are fatigue (3.8%) and headaches (2.0%) [212]. Pfizer modified the vaccines and presented four different vaccines for evaluation [213]. Children over the age of 16 started getting vaccinated on 11 December 2020 [214]. Studies have shown that this vaccine is less effective against variants, especially B.1.351, but it is effective against B.1.1.7 [215]. Among the adverse effects of vaccination, myocarditis, lymphadenopathy, and appendicitis were found [216]. Several studies have found that the risk of myocarditis is frequent in vaccinated patients and in patients infected with SARS-CoV-2 [216].

#### 10.2.2. Vaccine: mRNA-1273; Developer: Moderna/NIAID

The vaccine platform is mRNA encoding two proteins: stabilized S wrapped in lipid nanoparticles and the full-length target protein S, with two proline mutations (S-2P) to preserve the conformation of the prefusion. The trial protocol provided two doses of 100 μg for administrations (Table 9). From this, an efficacy of 94.1% emerged; therefore, the FDA approved the use of the vaccine in people over the age of 18 on 18 December 2020 [217]. The side effects registered are fever, muscles pain, and headache, especially after the second dose [218]. More common SEs are pain at the injection site (88.2%), erythema (8.6%), swelling (12.2%), and lymphadenopathy (14.2%) in slight or moderate forms, while a more severe reaction occurred in the mRNA-1273 group and after the second injection. The vaccine must be stored at −20 °C for 6 months, and after thawing, it can be stored for 30 days in a refrigerator at a temperature between 2 °C and 8 °C. The Moderna vaccine seems to be less effective against the B.1.351 South African, while it is effective against the B.1.1.7 English variant [215].

#### 10.2.3. Vaccine: AZD1222 (ChAdOx1 nCoV-19); Developer: Oxford University and AstraZeneca

The vaccine consists of a simian adenovirus vector defective in replication ChAdOx1 and the target is the codon-optimized S protein. The US studies are at Phase III in which participants receive two doses with AZD1222 or placebo. AZD1222 was authorized by the UK Medicines and Healthcare Products Regulatory Agency (MHRA), and the use of two doses of this vaccine was authorized at an interval of 4–12 weeks in adults over the age of 18 years (Table 10) [219]. This vaccine does not guarantee complete immunity but it can reduce symptoms after the infection. The vaccine is very easy to store since the adenovirus vaccine resists at least six months at refrigerator temperature [220]. Vaccines are hypothesized to immunize only the lower respiratory tract, which is protected by the IgG, while the upper respiratory tract is protected by the secretory IgA1 (sIgA1)) [170]. For this reason, these vaccines do not prevent the transmission of the virus, even if it can reduce the quantity [170]. On 30 January 2021, the Italian Agency for Drug Administration (AIFA) confirmed an efficacy of 59.5% in reducing COVID-19 symptoms and the benefit/risk ratio is favorable relative to the vaccine; thus, the use of the AstraZeneca vaccine was approved [221]. In March 2021, episodes of severe thrombosis occurred after vaccination and Denmark, Iceland, and Norway sus-pended the AstraZeneca vaccine [222]. In Italy, the Italian Agency of Drug (AIFA) suspended the use of batch number ABV2856 after three deaths [223]. Moreover, in Austria, the ABV5300 batch was suspended after the death of a woman. This lot was delivered to 17 countries, including Estonia, Lithuania, Luxembourg, and Latvia, which have also suspended it [224,225].

#### 10.2.4. Vaccine: Ad26.COV2-S; Developer: Janssen Pharmaceutical

The vaccine candidate Ad26.COV2 · S presents a protein S with a transmembrane domain and cytoplasmic tail. This protein has two mutations of the proline that stabilize the protein and allow the preservation of the pre-fusion conformation. Furthermore, the mutations allowed the removal of the polybasic site (cleavage site Ad26S.PP), which is subsequently named Ad26.COV2·S (Table 11) [179]. The Janssen vaccine, also named Ad.26.COV2S or JNJ-78436725, is a single dose COVID-19 vaccine realized by Johnson & Johnson and is indicated in people over the 18 years old of age. The efficacy of this vaccine against moderate-severe COVID-19 infection is 100%,while it is 85% in severe forms of the disease [226]. It is also easy to store and is stable at 2–8 °C for three months and 2 years at −20 °C [227]. The effectiveness is 66.9% after 14 days and 66.1% after 28 days against the Brazilian, British, and South African variants, while it is 57% against the South African variant, 501Y.V2 [185]. The Janssen vaccine was approved by the Food and Drug Ad-ministration (FDA) and it was authorized for public administration in the United States on February 27 [228] and in Europe on 11 March 2021 [229].

#### 10.2.5. Mucosal Vaccine Platform for COVID-19 Vaccine Development

The World Health Organization (WHO) stated that most COVID-19 vaccines have to be administered parenterally and intramuscularly, but the duration and effectiveness of the mucosal immune response in blocking the spread of the virus in the oral-respiratory tract is uncertain [230]. The enterocytes of the digestive mucosa show a high expression of ACE2, which is the SARS-CoV-2 receptor [231]. Interestingly, ACE2 is more present at the oronasal level than in the alveoli [232], suggesting a more intense viral replication in the oral and nasal mucosal than in alveoli [233]. From this evidence, the idea of an oral/nasal mucosal vaccine is derived.

#### 10.2.6. Vaxart Vaccine—Developer: Vaxart

Vaxart recently developed a COVID-19 oral recombinant vaccine that has entered into the first phase of study (NCT04563702). This vaccine is characterized by an adenoviral vector encoding the SARS-CoV-2 Spike genes “S” and nucleocapsid “N” proteins. This tablet dissolves in the digestive system, providing mucosal immunity against the virus [229]. Two weeks after the Vaxart vaccination in animals, a significant increase in neutralizing antibodies against SARS-CoV-2 was observed compared to the unvaccinated serum group [234].

#### 10.2.7. IosBio’s (Sabilitech’s) OraPro-COVID-19 Vaccine

The vaccine consists of a non-replicating viral vector with the “S” protein in a thermally stable capsulated form [235]. A modified adenovirus-5 (Adv5) vector encoding the “S” glycoprotein of the SARS-CoV-2 is encapsulated and orally administered in order to reach to the intestinal lymphoid tissues, inducing both cellular and humoral and cellular immune responses [236].

### 10.3. COVID-19 Vaccine and Antibody Response in Children

Pfizer and BioNTech have carried out a trial of their COVID-19 vaccine on children. The pediatric study, in children ages 5 to 11, is the first to reveal results in young children [237]. They used a lower dose of the vaccine because previous studies have shown that the adult dose could stress more side effects. The adults receive two 30-microgram doses of the vaccine three weeks apart. In school-aged children, this was reduced to 10 micrograms [237,238]. A study was conducted between preschool children (vaccine dose: 10 micrograms) and children between 16 and 25 years of age (adult vaccine dose) in which antibody responses and common side effects were evaluated [96]. The antibody response in children is similar to that found in the second group. Furthermore, the side effects such as fever and chills were similar to those in the second group who received the vaccine dose given to adults [237]. The COMIRNATY (COVID-19 Vaccine, mRNA) is an FDA-approved COVID-19 vaccine made by Pfizer for BioNTech licensing [239]:

- In individuals 16 years of age and older;
- In cases of emergency (EUA):
  - Prevent COVID-19 in individuals 12 through 15 years;
  - Administer a third dose to immunocompromised individuals over 12 years of age.

The US FDA has approved Pfizer Inc and BioNTech SE vaccines for preschool children (5–11 years), making it the first coronavirus vaccine in young children in the US [240]. The U.S. Center for Disease Control and Prevention has yet to provide advice on how to administer the vaccine, which will be decided after a group of external consultants discuss the plan. Vaccines for young children have only been authorized in China, the United Arab Emirates, and Cuba. The FDA has cleared a dose of 10 micrograms of the Pfizer vaccine in young children and less than 30 micrograms of the original vaccine for those 12 years of age and older. Vaccine manufacturer Comirnaty said that the vaccine is 90.7% effective against coronavirus in children aged 5 to 11 [240].

### 10.4. COVID-19 Vaccines for Moderately to Severely Immunocompromised People

In general, the immunocompromised patients represent almost 3% of the adult population. The CDC recommends that people with moderate to severely compromised immune systems receive an additional dose of COVID-19 mRNA vaccines at least 28 days after a second dose of the Pfizer-BioNTech COVID-19 vaccine or Moderna COVID-19 vaccine [241]. Studies reported that immunocompromised subjects develop lower immunity after vaccination than healthy subjects. Therefore, an additional dose of the vaccine is thought to provide protection against COVID-19 infections [84]. In other studies, it was reported that immunocompromised people, even if vaccinated, had symptoms that required hospitalization. Therefore, such individuals were more likely to become infected and transmit the virus [242]. Currently, the CDC recommends administering a third dose to individuals with moderate/severe immune system impairment. This includes the following [241]:

- People being treated for cancer;
- People being treated with immunosuppressive drugs following organ transplantation;
- People being treated with immunosuppressive drugs following stem-cell transplantation (within 2 years);
- People with immunodeficiency as a result of syndromes such as DiGeorge syndrome and Wiskott–Aldrich syndrome;
- People with advanced or untreated HIV infections;
- Patients with suppressed immune responses following high-dose corticosteroid therapy.

## 11. Vaccine’s Implications in Pregnancy and Fertility

Pregnant women with COVID-19 have a higher risk of developing gestation complications; therefore, the opportunity to be vaccinated is important [243,244]. Women should be encouraged to complete their vaccination before conception [245,246]. Vaccinating pregnant women ensures protection for immunologically immature infants [247]. After maternal mRNA COVID-19 vaccination, antibodies are transferred through the placenta to the newborn [247]. A recent study showed the effect of these antibodies [247]. A women received two doses of the BNT162b2 vaccine during her second trimester, and the baby was born preterm as a consequence of maternal complications. The persistence of anti-SARS-CoV-2 S antibodies in the infant at 2 weeks, 6 weeks, 3 months, and 6 months of age has been observed [247]. For mRna vaccines, the mRna does not enter in the cell nucleus and has an estimated half-life of 8–10 h. Given the short half -life of mRna vaccines, it is unlikely to be transmitted during pregnancy or to the infant during breastfeeding. If the mRna vaccine enters breast milk, it is expected to be broken down during the digestive process [248]. In a recent study, 37 milk samples and 70 breast swabs (before and after breast washing) have been collected from 18 women recently diagnosed with COVID-19. Samples were analyzed for SARS-CoV-2 RNA using RT-qPCR [249]. The ELISA test was used to analyzed milk; in particular, it was analyzed for the following: IgA and IgG specific for the nucleocapside protein, receptor-binding domain (RBD), S2 subunit of the Spike protein of SARS-CoV-2, and for its ability to neutralize SARS-CoV-2 [249]. The hypothesis of the transmission of COVID- 19 infection through breast milk is not supported by the results of this research [249]. Milk produced by women with COVID-19 is a source of antibodies (anti-SARS-CoV-2 IgG and IgA); for this reason, recent students suggest continuing breastfeeding during maternal COVID-19 illness [249,250,251,252,253,254,255]. The mRna COVID-19 vaccines have been shown to be immunogenic based on the evaluation of both humoral and cellular immune responses in pregnant, lactating, and non-pregnant women [256]. After the second dose of mRna vaccines, 13% of pregnant women and 47% of non-pregnant women showed fever. There are consistent data to validate that vaccination causes higher antibody responses than infection [256,257]. The detection of binding and neutralizing antibodies in the newborn’s cord blood suggests an efficient transplacental transfer of maternal antibodies [257]. A study has been carried out on six pregnant women with COVID-19 infections admitted from February to March 2020 at the Zhongan Hospital of Wuhan University [258]. In five infants, IgG concentrations were elevated. IgM, because of its larger macromolecular structure, is not usually transferred across the placenta; for this reason, it was detected only in two infants [259]. In a study, the placentas of six women who were convalescing from SARS-CoV infections in the third trimester of pregnancy have been analyzed. The placentas of two women had abnormal weights and pathologies, and the causes of these effects are unknown [260]. Alternatively, if the virus crossed the placenta, IgM could be produced by the newborn [260]. A recent study analyzed, in 84 women voluntarily vaccinated against COVID-19, whether maternal immunization caused the secretion of SARS-CoV-2 antibodies in breast milk and could result in potential adverse events for women and their children [261]. Six weeks after vaccination, the secretion of specific SARS-CoV-2 IgA and IgG antibodies in breast milk has been observed, and the data document the secretion of IgA after 2 weeks of vaccination and a peak of IgG after 4 weeks. Antibodies found in this women’s breast milk have a protective effect against infections in the newborn. The mRna COVID-19 vaccines (Pfizer/Biontech Bnt 1262b2 and Moderna mrna-1273) have so far been shown to be safe and effective, but in an early stage of use, they encountered some resistance based on presumed adverse effects on fertility [262]. This led a research group to carry out and publish a prospective study on the evaluation of sperm parameters before and after the administration of an mRNA vaccine. A total of 45 volunteers between the age of 18 and 50 have been enlisted and semen samples have been provided before vaccination and 70 days after the second dose. The samples have been measured for the following: sperm volume and concentration, sperm motility, and total mobile sperm count (TMSC) [263]. Forty-six point seven percent (46.7%) of the forty-five (45) volunteers received Pzier/Biontech vaccine and fifty-three point three percent (53.3%) received Moderna. Results showed sperm concentrations and a basal TMSC of 26 million/mL and 36 million, respectively. After the second vaccine dose, the median sperm concentration increased to 30 million/mL (*p* = 0.02) and TMSC to 44 million (*p* = 0.01), and there was a significant increase in sperm volume and sperm motility. No case of azoospermia has been documented after vaccination [263]. Despite the limitations of the study generated by the lack of a control group and the fact that semen analysis is an imperfect predictor of male fertility, these data make it unlikely that these vaccines, containing mRna and not the virus, adversely affect sperm parameters [263].

## 12. Side Effects and Post-Vaccination Pathologies Related to Antibody Titers

Side effects can be divided into local ones, such as swelling, redness, pain and regional lymphadenopathy, palms, and soles itchiness, and systemic symptoms, such as myalgia, headache, fatigue, fever, arthralgia, chills, nausea, diarrhea, and vomiting [264]. In the literature, they were reported cases of myocarditis after vaccination [265,266,267] as being potential neurological disorders [268]. Therefore, urological complications have also been reported, although they are rarer [269]. However, since they are common in the general population, there should be no correlation between them and vaccinations. There are also documented cases of the reactivation of Herpes Zoster [270], breast implant seroma [271], cardiac arrest, cerebral venous sinus thrombosis [272], pulmonary embolism, nephrotic syndrome, acute kidney injury (AKI) [273], central serous retinopathy [274], and Turner syndrome [275]. Side effects in orofacial region are rare and included acute peripheral facial paralysis (Bell’s palsy), facial swelling, and swelling of the lips, face, or tongue associated with anaphylaxis [276]. In various studies, it was shown that liver, kidney, and heart transplant recipients have a low immune response to the BNT162b2 SARS-CoV-2 vaccine; therefore, they remain at a high risk for COVID-19 [277,278,279]. Previous cases of this are from the flu shot. COVID-19 vaccinations are associated with multiple sclerosis [51,52], Still’s disease [280], autoimmune hepatitis [281,282], Guillain–Barre syndrome [283], and thromboembolytic episodes called vaccine-induced prothrombotic immune thrombocytopenia. VIPIT is caused by platelet activation by antibodies, leading to thrombosis and subsequent thrombocytopenia similar to thrombocytopenic purpura or heparin-induced thrombocytopenia [284,285]. In an analysis that includes persons with a history of cancer, the beginning of axillary and supraclavicular lymphadenopathy and ipsilateral to the injection site with Pfizer/BioNTech BNT162b2 mRNA, AstraZeneca ChAdOx1, and Moderna mRNA-1273 vaccines is reported [286,287]. In addition to the anti-COVID-19 vaccines, lymphadenopathy was also detected in addition to the anti-COVID-19 vaccines, lymphadenopathy was also detected previously later smallpox, Bacille Calmette–Guerin (BCG), human papillomavirus (HPV), and H1N1 influenza A virus vaccines [268,270,271]. Autoimmune reactions after vaccination are infrequent (less than 0.01% of all recipients), most cases are asymptomatic, or mild autoimmune diseases are triggered by an insufficient response by intrinsic homeostatic systems against the secretion of autoantibodies. They are formed by molecular mimicry even though some theories speak of the formation of immune complexes that recreate an imbalance of T lymphocytes and a bystander activation because of an exorbitant immune defence to vaccine adjuvants [10,288,289,290,291,292,293,294,295,296,297,298]. In a study of five multiple sclerosis patients receiving B-cell depleting treatments, although there was an absence or the very low concentration of anti-S antibodies, a T-cell response was observed. The interferon (IFN)-γ release assay (IGRA) is very important for detecting T-cell titers: This can be an additional diagnostic tool against SARS-CoV-2 infection evaluating the immunological defence after natural infection or vaccination [291,299,300,301,302,303,304,305]. Aside from genetics and comorbidities, aging causes immunosenescence and inflammaging. This aspect seems to produce an immunological disability against infections and reactions to vaccines [306]. Actually, in an analysis, it was observed that, in the 20–40 age range, there is an incremental increase in antibodies that is shorter than in older people, suggesting a priority vaccination for this category. In particular, there is a greater likelihood of high IgG levels in females than in males [307,308]. A study hinted that obesity presented a decreased rate of Breg cells, which leads to augmented titers of Th1/Th17 cytokines. There are several hypotheses about the causes of a reduced antibody response in obese children and adolescents following tetanus vaccination: Mechanical causes refer to low absorptions due to excess adipose tissue; other causes are a low vaccine dose in relation to weight or a low antibody response because of the chronic low grade inflammation expressed by the greater titers of IL-6 [309,310,311]. On the other hand, very vulnerable dialysis patients, after the vaccine, have antibody titers that can protect them from infection [312]. In a study with hematological malignancies, it was show that half of the persons achieved immunity after vaccination. There was a correlation between T cells with anti-S IgG levels after six weeks; in fact, some patients with B-cell lymphopenia and no humoral responses still achieved a T-cell response, suggesting that in these subjects with partial immunity due to a lack of humoral immunity, T cells play a protective role [313]. In a rheumatoid arthritis study, a low antibody response to vaccine was observed in patients treated with immunosuppressive drugs [314]. In general, there is a high antibody response in patients with solid tumors or who have passed COVID-19 tests previously. Instead, there is a low response in patients with haematological malignancies, anti-CD20 antibody treatment, stem cell transplant, and cart-T cell therapy [315]. Persons who had COVID-19 before vaccination had more side effects than naive participants, but they were not serious. In addition, the side effects after the first dose are greater than with the second, although they were not more severe [316,317]. In an immunology study on rhesus macaques, an induction of TFH cells for the maturation of the germinal centers (GC) was reported, formed by activated B cells that undergo clonal expansion and selection within the secondary lymphoid organs, and the activation of humoral immunity with the secretion of antibody-secreting cells (ASCs) and broadly neutralized antibodies (bNAbs) after vaccination. In particular, GC reactions have an important aim in improving the quality of specific antibodies against viremia [318,319]. The aim of TFH cells can also be highlighted by other studies in which it was noted that, in certain COVID-19 persons who have high levels of TNF-a, they block the last phase of the differentiation of follicular T helper cells, which become defective. In addition to this, there is an absence of the germinative centers (GC) [320]. Cytotoxic T cells play the aim of destroying virus-infected cells, protecting against the further spread of infection. It can be added that helper T cells are important for B cell function in the initial secretion of antibodies and their maintenance over time [321]. Apart from the memory of T and B cells, it has been shown how neutralized antibodies have a concrete influence on a protective role against infection [322]. However, in other studies, there has not been a long response from the Nabs [323]. A highly significant inter-correlation was noted between total Ig anti-RBD and IgG anti-S1/S2 and IgG anti-RBD. Titers of neutralizing antibodies will be important to support an optimal degree of protection against SARS-CoV-2 infection and any variants. Therefore, the detection of these antibodies (especially IgG and IgA) would help improve vaccine formulations to enhance their titer against the new strains [324]. Even if the viral infection creates a more intense response than the first dose of vaccine, it has been noted that after the second, the antibody titers are higher and longer lasting versus COVID-19 [325,326]. Vaccination induces a stronger antibody response in subjects who have passed COVID-19 and a weaker response in asymptomatic subjects than in febrile subjects [327]. Antibodies response with mRNA-1273 vaccine persisted 180 days after the second dose, as identified by three distinct serologic assays [327]. In one study, an antibody reduction of 37.9% was observed in seronegative persons and 44.7% in seropositive subjects compared to the highest antibody titer [328,329,330,331,332,333,334,335,336,337]. In an important study, it was shown that 30% of the vaccinated elderly did not reach the antibody response after the second dose. In addition, the study was able to state that there is no correlation between the production of neutralizing antibodies or anti-s IgG and the presence or absence of postvaccination side effects [338]. There are no associations between the blood group or influenza vaccination as protective factors against SARS-CoV-2 infection [339]. In one study, the correlation between age and BMI was confirmed in the different antibody response after vaccination, while gender had a lower significance [340]. Given the role of vitamin D in lowering pro-inflammatory cytokines and increasing anti-inflammatory ones, its deficiency leads to systemic worsening in patients with COVID-19. Therefore, it can be hypothesized that a vitamin D deficiency may lead to a reduced antibody response to the vaccine [15,87,341,342,343,344,345].

## 13. Discussion

Understanding the mechanisms would help avoid hospitalizations, intensive care, and deaths from SARS-CoV-2. Some hypotheses have emerged from recent genomic studies [159,160,161,162]. In fact, these studies have revealed two important facts. The first study shows that men under 60 years of age with a severe or lethal form of COVID-19, about 3.5%, have mutations in the TLR7 gene at a rate of more than 1%, and these are genes that intervene with the production and operation of the entire chain linked to the interferons, resulting in a lack of this immediate molecule that enables the virus to destroy or slow down its action. Thus, a therapeutic pathway for patients who lack these molecules was thought to be that of using interferons [159]. The second study showed that there are 10% of patients with a severe course who show a production of autoantibodies against type I interferon (IFN-1), and most of them are over 80 years old. There are several types of interferons, all of which are particularly important in the innate immune response. All 17 subtypes of type I IFN bind to the same heterodimeric receptor (IFNAR1 and IFNAR2), including 13 subtypes of IFN-α, IFN-ω, IFN-β, IFN-ε, and IFN-κ [346,347]. Those that are most phylogenetically related are the 13 subtypes of IFN-α and IFN-ω, whereas IFN-β, IFN-ε, and IFN-κ are more distantly related [346]. Auto-Abs for IFN-α2 and/or IFN-ω were found mainly in men (95%) and elderly people (half of the patients with antibodies were older than 65 years). This would explain why men have a higher risk of developing severe forms of COVID-19. A study was performed with sensitive immunoassays and neutralisation tests detecting the presence of autoantibodies against type I interferons α, β, or ω in plasma samples from a large cohort of patients with COVID-19 and prepandemic controls [348]. Neutralising auto-Abs at high concentrations of IFN-α and/or IFN-ω are present in 0.18% of individuals between 18 and 69 years, 1.1% between 70 and 79 years and 3.4% >80 years, and in all individuals with a severe disease course but in none of the tested individuals with asymptomatic or paucisymptomatic infections. The percentage of patients younger than 40 years with auto-Abs was 9.6%, while those over 80 years comrpised more than 21%. The proportion of male patients over 80 years with critical COVID-19 carrying IFN-α2 and/or IFN-ω neutralising auto-Abs (100 pg/mL) increased to 23%. It appears that at least 18% of patients dying of COVID-19 pneumonia produce auto-Abs capable of neutralising type I IFNs (100 pg/mL) in plasma 1:10 [346]. In contrast, IFN-β-neutralising auto-Abs do not become more frequent with age and their presence was associated with critical but not significantly severe diseases [348]. Usually, the production of antibodies against interferon occurs in patients with autoimmune diseases, which, in the study, resulted in only three in the 10% of cases. For the treatment plan of these patients, the picture changes, because the use of interferons would trigger a massive and severe inflammatory response. A new clinical protocol, to be approved and tested, has been devised using plasmapheresis to eliminate autoantibodies from the blood [348]. The interferon, a molecule produced by our bodies in response to infection, plays a key role in both mechanisms. Other findings indicate that about 20% of all COVID-19 victims produce autoantibodies directed against NFI type I, a rare acquired immunodeficiency disease. By evaluating both of these, regardless of age, about 15% of the population had a defect in the interferon synthesis pathway, either because of the mutation or because it was destroyed by the production of autoantibodies [162]. Professor Giuseppe Novelli, the director of the Laboratory of Medical Genetics at the University of Rome Tor Vergata, says there are genetic reasons behind it, and that in these high-risk and fatal COVID-19 subjects, an in-depth study should be carried out on HLA genes (the human anti-leukocyte gene), which does not recognise its own genetic heritage. Therefore, priority could be given to vaccinating the population in which these two data are found (the presence of anti-IFN-1 autoantibodies or the presence of mutations in the interferon genes) [346,349]. In another retrospective study, autoimmune antibodies for common antigens were analysed in 115 COVID-19 hospitalised patients with different degrees of severity: an erythrocyte lysate, lipid phosphate-dylserine (PS), and DNA [350]. A high titer of IgG autoantibodies against erythrocyte lysates was found in up to 36% of patients. Anti-DNA and anti-PS antibodies with a positive predictive value of 85.7% and 92.8%, respectively, correlated with patients who subsequently had a severe disease development. Of the total severe cases, 24% showed at least one of the two autoantibodies. The presence of anti-DNA antibodies correlated strongly with the presence of markers of cell damage, coagulation, neutrophil levels, and erythrocyte size [350,351]. Thus, the presence of anti-DNA and anti-PS autoantibodies may play an important role in the pathogenesis of COVID-19 and could be sought as a predictive biomarker for disease severity and specific clinical manifestations. Another study also correlated the presence of autoantibodies against the lung protective protein Annessin-A2 with the mortality of COVID-19 patients [352,353]. The innate immune response is non-specific, because it is not influenced by previous exposure to other pathogens. Phagocytes, such as macrophages and neutrophils, and Natural Killer (NK) cells intervene in this first response of the immune system, with n the first hours of an infection, by attacking pathogens. The first phase of the inflammatory process is, thus, initiated, characterised by a cascade of cytokines accompanied by pain, redness, heat, swelling, and vasodilation [354]. The adaptive immune response, on the other hand, intervenes in a specific manner and with a memory capacity to defend the body effectively against the various species of viruses and bacteria to which it is exposed [355]. Lymphocytes, both T- and B-class, intervene differently in this second phase of the immune response [356]. T lymphocytes are attracted by dendritic cells and activated by antigens (proteins present on the surface of the virus), which are recognised as a danger and eliminated by their receptors. T lymphocytes (CD4 and CD8) responsible for “protective cellular immunity” activate the cell-mediated immune reaction, while B lymphocytes are responsible for the massive and differentiated production of antibodies (class M, A, D, E, and G immunoglobulins) as humoral immunity, which lasts over time, with a high antibody titer and sterilising immunity that would prevent a second infection [357,358,359]. Class M immunoglobulins (IgM) are the first to be produced in response to an infection, giving way to the production of IgG (IgG is the most common antibody in the blood). The production of antibodies provides the immunological memory that enables the immune system to recognise pathogens it has come into contact with [360]. The presence and production of antibodies have been the bases for the development of serological tests and vaccines against viruses and bacteria [357]. In the mechanism of innate immunity, the first stage of defence against a pathogen, an interferon plays a fundamental role, which is also recognised in the defence against SARS-CoV-2 [346]. Thus, genomic mutations at this level may place the individual at greater risks of developing a severe form of the disease. Elderly groups have been shown to have a more severe course of the disease caused by SARS-CoV-2 due to the immunosenescence phenomenon, which results in changes in both innate and adaptive immunity [361,362]. The elderly exhibit an uncontrolled activation of the innate immune response that determines the cytokine cascade and tissue damage. The inability to activate an adaptive immune response associated with a heightened pro-inflammatory state does not slow down viral replication or the likely clinical consequences initiated by a cytokine storm that produces an endothelial injury and disseminated organ lesions [363,364]. SARS-CoV-2 is a virus that profoundly alters the immune response due to the intense production of cytokines, reducing cellular immunity and inhibiting the protective response. In many patients over 60 years of age, a reduction or even disappearance of cytotoxic CD8 lymphocytes, lymphocytes capable of killing virus-infected cells and conferring cellular immunity, has been observed [365]. When the virus penetrates the respiratory (and intestinal) epithelium, it replicates with the production of Interleukin-1 beta (IL-1beta) and In-terleukin-18 (IL-18), two cytokines produced by the immune system that can exacerbate the state of inflammation linked to virus pyroptosis, and this induces epithelial cell death [366]. In some patients, inflammation occurs with the production of T-cells (CD4 and CD8) and neutralising antibodies, resulting in a protective response [367,368]. In other patients, particularly older ones, a neutrophilic infiltrate prevails. CD4 lymphocytes begin to produce large quantities of cytokines, which amplify the inflammation. CD4 and CD8 cells become exhausted and the patient becomes seriously ill [361]. Currently, the CHGE Consortium is studying a population of 150 super-resistant individuals who, despite having been in prolonged and close contact with SARS-CoV-2-infected patients, have neither become infected nor were virus-specific antibodies detected in various tests [367]. It is assumed that the resistance of these individuals to virus entry is due to monogenic variations [358]. Recently, researchers Zeberg and Pääbo associated a reduction of about 22% in the risk of becoming severely ill with COVID-19 when infected with SARS-CoV-2 in those with a haplotype on chromosome 12. This haplotype is inherited from Neanderthals. It is found in all regions of the world, at 25–30% in most populations of Eurasia, but it is almost completely absent in African populations south of the Sahara and encodes important proteins during RNA virus infections [359]. In contrast, a recent study identified two genomic regions associated with severe COVID-19: a region on chromosome 3 that contains six genes and is present in 65% in South Asia and about 16% in Europe while it is almost absent in East Asia and a region on chromosome 9 that determinates ABO blood groups [369,370,371,372].

## Figures and Tables

**Table 1:** Currents variants of concern (classification from WHO website).

Codification	Vector Lineages	Clades	Nextstrain Clades	Aminoacid Changes	Emerging Regions and Period of Strain Detection	Date of First Detection
Alpha	B.1.1.7	GRY	20I.V1	+S: 484K +S: 452R	United Kingdom, Sep-2020	18-Dec-2020
Beta	B.1.351	GH/501Y.V2	20H.V2	+S: L18F	South Africa, May-2020	18-Dec-2020
Gamma	P.1	GR/501Y.V3	20J.V3	+S: 681H	Brazil, Nov-2020	11-Jan-2021
Delta	B.1.617.2	G/478K.V1	21; 21I; 21J	+S: 417N +S: 484K	India, Oct-2020	VOI: 4-Apr-2021 VOC: 11-May-2021
Omicron	B.1.1.529 (Subvariants: BA 1, BA 2, BA 3, BA 4 and BA 5)	GR/484A	21K	-+S: L452R -+S: F486V -+S: R493Q	Multiple countries, Nov-2021	VUM: 24-Nov-2021 VOC: 26-Nov-2021 Sub BA 4/5: January 2022

**Table 2:** Currents variants of interest (classification from WHO website).

Codification	Vector Lineages	Clades	Nextstrain Clades	Emerging Regions and Period of Strain Detection	Date of First Detection
Lambda	C.37	GR/452Q.V1	21G	Peru, Dec-2020	14-Jun-2021
Mu	B.1.621	GH	21H	Colombia, Jan-2021	30-Aug-2021

**Table 3:** Currents variants under monitoring (classification from WHO website).

Vector Lineages	Clades	Nextstrain Clades	Emerging Regions and Period of Strain Detection	Date of First Detection
R.1	GR	-	Multiple countrie Jan-2021	07-Apr-2021
B.1.466.2	GH	-	Indonesia, Nov-2020	28-Apr-2021
B.1.1.318	GR	-	Multiple countries, Jan-2021	02-Jun-2021
B.1.1.519	GR	20B/S.732A	Multiple countries, Nov-2020	02-Jun-2021
C.36.3	GR	-	Multiple countries, Jan-2021	16-Jun-2021
B.1.214.2	G	-	Multiple countries, Nov-2020	30-Jun-2021
B.1.427 B.1.429	GH/452R.V1	21C	United States of America, Mar-2020	VOI: 5-Mar-2021 VUM: 6-Jul-2021
B.1.1.523	GR	-	Multiple countries, May-2020	14-Jul-2021
B.1.619	G	20A/S.126A	Multiple countries May-2020	14-Jul-2021
B.1.620	G	-	Multiple countries, November 2020	14-Jul-2021
C.1.2	GR	-	South Africa, May 2021	01-Sep-2021
B.1.617.1	G/452R.V3	21B	India, Oct-2020	VOI: 4-Apr-2021 VUM: 20-Sep-2021
B.1.526	GH/253G.V1	21F	United States of America, Nov-2020	VOI: 24-Mar-2021 VUM: 20-Sep-2021
B.1.525	G/484K.V3	21D	Multiple countries, Dec-2020	VOI:17-Mar-2021 VUM: 20-Sep-2021
B.1.630	GH	-	Dominican Republic, Mar-2021	12-Oct-2021

**Table 5:** Coronavac SINOVAC vaccine general characteristics.

Coronovac Sinovac Vaccine	
General Characteristics	
Type of Vaccine	Inactivated SARS-CoV-2 Virus Inactivating Agent: β-propiolactone
Storage temperature	2–8 °C
Vaccine Administration and dosages	Two doses 0.5 mL (~14 days)
Phase III Efficacy rate	50.4%
Variant Efficacy	B.1.1.7 B.1.351; P1

**Table 6:** Sputnik V Vaccine general characteristics.

Sputnik V Vaccine	
General Characteristics	
Type of Vaccine	dsDNA Vaccine Adenovirus Delivered AD26/AD5
Storage temperature	Long-Term Storage (2 years): −18 °C Short-Term Storage (3 month): 2–8 °C
Vaccine Administration and dosages	Two doses 0.5 mL (~28 days)
Phase III Efficacy rate	91%
Variant Efficacy	B.1.1.7 B.1.351; P1

**Table 7:** Vaccine typologies for SARS-CoV-2.

PLATFORM	VACCINE	DEVELOPER
RNA vaccine	BNT162	Pfizer/BioNTech/Fosun
RNA vaccine	Spikevax (COVID-19 Vaccine Moderna)	Moderna/NIAID
Non-replicating viral vector	Vaxzevria ChAdOx1-S	Astrazeneca AB
Non-replicating viral vector	Ad26.COV2-S	Janssen-Cilag International NV

**Table 8:** Pfizer/BioNTech vaccine general characteristics.

BNT162b2 Pfizer/BioNTech Vaccine	
General Characteristics	
Type of Vaccine	mRNA Vaccine Lipid Nanoparticles Delivered
Storage temperature	Long-term Storage (6 month): −70 °C Short-Term Storage (5 days): 2–8 °C
Vaccine Administration and dosages	Two doses 0.3 mL (~21 days)
Phase III Efficacy rate	95%
Variant Efficacy	B.1.1.7; B.1.135; P1; B.1.429; B.1.617.2

**Table 9:** Moderna vaccine general characteristics.

m-RNA-1273 Moderna/NIAID Vaccine	
General Characteristics	
Type of Vaccine	mRNA Vaccine Lipid Nanoparticles Delivered
Storage temperature	Long-Term Storage (6 month): −20 °C Short-Term Storage (30 days): 2–8 °C
Vaccine Administration and dosages	Two doses 0.5 mL (~28 days)
Phase III Efficacy rate	94.5%
Variant Efficacy	B.1.1.7; B.1.135; P.1; B.1.429 B.1.617.2

**Table 10:** AstraZeneca vaccine general characteristics.

AZD1222 (ChAdOx1 nCoV-19) Astrazeneca Vaccine	
General Characteristics	
Type of Vaccine	dsDNA Vaccine Adenovirus Delivered
Storage temperature	about 2–8 °C (6 month)
Vaccine Administration and dosages	Two doses 0.5 mL (12 weeks)
Phase III Efficacy rate	81.3%
Variant Efficacy	B.1.1.7 B.1.135 P.1B.1.429 B.1.617.2

**Table 11:** Janssen vaccine general characteristics.

Ad26.COV-S Janssen Pharmaceutical Vaccine	
General Characteristics	
Type of Vaccine	dsDNA Vaccine Adenovirus Delivered
Storage temperature	Long-Term Storage (2 years): −20 °C Short-Term Storage (3 month): 2–8 °C
Vaccine Administration and dosages	Single dose 0.5 mL
Phase III Efficacy rate	66%
Variant Efficacy	72% B.1.1.7. 57% B.1.135 & P.1 B.1.617.2

## Data Availability

All experimental data to support the findings of this study are available from the corresponding authors upon request.

## List of Abbreviations

ACE2	angiotensin-converting enzyme-2
ACE	angiotensin-converting enzyme
ACE1	angiotensin-converting enzyme 1
AIFA	Agenzia Italiana del Farmaco
Alpha	English variant B.1.1.7
Anti-RBD IgG	Immunogloublin G anti receptor-binding domain
Anti-Spike	test igg anti-Spike
BAU	unità arbitrarie vincolanti
Beta variant	(former of South Africa)
BMI	body mass index
CI	intervallo di confidenza
CLIAs	chemiluminescence immunoassay
CRP	C-reactive protein
Delta	Indian variant B.1.617.2
ELISA	enzyme-linked immunosorbent assay
EMA	European Medicines Agency
ETA	variant B.1.525; date of designation Mar-2021
Gamma	Brasilian variant P.1
hACE2 receptor	human angiotensin I-converting enzyme 2 receptor
IFN	Interferon
IgA	Immunoglobulins A
IgG	Immunoglobulins G
IgM	Immunoglobulins M
IOTA	variant B.1.526; earliest documented samples USA (Nov-2020), date of designation Mar-2021
IQR	interquartile range
KAPPA	Indian variant B.1.617.1
LAMBA	variant C.37; earliest documented samples Peru (Aug-2020), date of designation June 2021
LFIAs	lateral flow immunoassays.
MERS	Middle East Respiratory Syndrome
MMF	mycophenolate mofetil
MPA	mycophenolic acid
MPPDH	inosine-5′-monophosphate dehydrogenase
NAAT	nucleic acid amplification test
NGS	Next-Generation Sequencing
bNAbs	Broadly neutralizing antibodies
N-IgG	anti-N-IgG
PRD	viral prion-like domain
RBD	receptor-binding domain
RBDs	receptor-binding domains
RDB-IgG	receptor-binding domain neutralizing antibodies
RT-PCR	real-time PCR Polymerase chain reaction
S	Spike glycoprotein
SARS-CoV-1	Severe Acute Respiratory Syndrome Coronavirus 1
SARS-CoV-2	Severe Acute Respiratory Syndrome Coronavirus 2 (COVID-19)
SARSr-CoV Rp3	salivar protein similar to fused 8a and 8b SARS-CoV Beta Coronavirus
S-IgG	anti-Spike IgG
thio-NAD	thionicotinamide-adenine dinucleotide
TNF	tumor necrosis factor
VIPIT	prothrombotic immune thrombocytopenia
VOC	variants of concern
VOI	variants of interest
ZETA	variant P.2

## References

[1] Cao W., Fang Z., Hou G., Han M., Xu X., Dong J., Zheng J. (2020). The Psychological Impact of the COVID-19 Epidemic on College Students in China. Psychiatry Res..

[2] CDC CDC 2020-2021 Flu Vaccine Campaign Kickoff. https://www.cdc.gov/flu/spotlights/2020-2021/2020-21-campaign-kickoff.htm.

[3] Han Q., Lin Q., Ni Z., You L. (2020). Uncertainties about the Transmission Routes of 2019 Novel Coronavirus. Influenza Other Respir. Viruses.

[4] Klompas M., Baker M.A., Rhee C. (2020). Airborne Transmission of SARS-CoV-2: Theoretical Considerations and Available Evidence. JAMA.

[5] Jiang F., Deng L., Zhang L., Cai Y., Cheung C.W., Xia Z. (2020). Review of the Clinical Characteristics of Coronavirus Disease 2019 (COVID-19). J. Gen. Intern. Med..

[6] Spagnolo L., Vimercati L., Caputi A., Benevento M., De Maria L., Ferorelli D., Solarino B. (2021). Role and Tasks of the Occupational Physician during the COVID-19 Pandemic. Medicina.

[7] Boffetta P., Violante F., Durando P., De Palma G., Pira E., Vimercati L., Cristaudo A., Icardi G., Sala E., Coggiola M. (2021). Determinants of SARS-CoV-2 Infection in Italian Healthcare Workers: A Multicenter Study. Sci. Rep..

[8] Vimercati L., De Maria L., Quarato M., Caputi A., Stefanizzi P., Gesualdo L., Migliore G., Fucilli F.I.M., Cavone D., Delfino M.C. (2021). COVID-19 Hospital Outbreaks: Protecting Healthcare Workers to Protect Frail Patients. An Italian Observational Cohort Study. Int. J. Infect. Dis..

[9] Luyten J., Beutels P. (2016). The Social Value Of Vaccination Programs: Beyond Cost-Effectiveness. Health Aff..

[10] Scarano A., Inchingolo F., Lorusso F. (2020). Facial Skin Temperature and Discomfort When Wearing Protective Face Masks: Thermal Infrared Imaging Evaluation and Hands Moving the Mask. Int. J. Environ. Res. Public Health.

[11] Charitos I.A., Del Prete R., Inchingolo F., Mosca A., Carretta D., Ballini A., Santacroce L. (2020). What We Have Learned for the Future about COVID-19 and Healthcare Management of It?. Acta Biomed..

[12] Dohan Ehrenfest D.M., Del Corso M., Inchingolo F., Charrier J.-B. (2010). Selecting a Relevant in Vitro Cell Model for Testing and Comparing the Effects of a Choukroun’s Platelet-Rich Fibrin (PRF) Membrane and a Platelet-Rich Plasma (PRP) Gel: Tricks and Traps. Oral Surg. Oral Med. Oral Pathol. Oral Radiol. Endod..

[13] Scarano A., Inchingolo F., Lorusso F. (2020). Environmental Disinfection of a Dental Clinic during the COVID-19 Pandemic: A Narrative Insight. Biomed. Res. Int..

[14] Scarano A., Inchingolo F., Rapone B., Festa F., Tari S.R., Lorusso F. (2021). Protective Face Masks: Effect on the Oxygenation and Heart Rate Status of Oral Surgeons during Surgery. Int. J. Environ. Res. Public Health.

[15] Inchingolo A.D., Inchingolo A.M., Bordea I.R., Malcangi G., Xhajanka E., Scarano A., Lorusso F., Farronato M., Tartaglia G.M., Isacco C.G. (2021). SARS-CoV-2 Disease Adjuvant Therapies and Supplements Breakthrough for the Infection Prevention. Microorganisms.

[16] Satarker S., Nampoothiri M. (2020). Structural Proteins in Severe Acute Respiratory Syndrome Coronavirus-2. Arch. Med. Res..

[17] Yadav R., Chaudhary J.K., Jain N., Chaudhary P.K., Khanra S., Dhamija P., Sharma A., Kumar A., Handu S. (2021). Role of Structural and Non-Structural Proteins and Therapeutic Targets of SARS-CoV-2 for COVID-19. Cells.

[18] Balzanelli M.G., Distratis P., Lazzaro R., D’Ettorre E., Nico A., Inchingolo F., Dipalma G., Tomassone D., Serlenga E.M., Dalagni G. (2022). New Translational Trends in Personalized Medicine: Autologous Peripheral Blood Stem Cells and Plasma for COVID-19 Patient. J. Pers. Med..

[19] Huang Y., Yang C., Xu X.-F., Xu W., Liu S.-W. (2020). Structural and Functional Properties of SARS-CoV-2 Spike Protein: Potential Antivirus Drug Development for COVID-19. Acta Pharm. Sin..

[20] Yang J., Petitjean S.J., Koehler M., Zhang Q., Dumitru A.C., Chen W., Derclaye S., Vincent S.P., Soumillion P., Alsteens D. (2020). Molecular Interaction and Inhibition of SARS-CoV-2 Binding to the ACE2 Receptor. Nat. Commun..

[21] Bošnjak B., Stein S.C., Willenzon S., Cordes A.K., Puppe W., Bernhardt G., Ravens I., Ritter C., Schultze-Florey C.R., Gödecke N. (2021). Low Serum Neutralizing Anti-SARS-CoV-2 S Antibody Levels in Mildly Affected COVID-19 Convalescent Patients Revealed by Two Different Detection Methods. Cell. Mol. Immunol..

[22] Arashkia A., Jalilvand S., Mohajel N., Afchangi A., Azadmanesh K., Salehi-Vaziri M., Fazlalipour M., Pouriayevali M.H., Jalali T., Mousavi Nasab S.D. (2021). Severe Acute Respiratory Syndrome-coronavirus-2 Spike (S) Protein Based Vaccine Candidates: State of the Art and Future Prospects. Rev. Med. Virol..

[23] Tracking SARS-CoV-2 Variants. https://www.who.int/en/activities/tracking-SARS-CoV-2-variants/.

[24] Chavda V.P., Apostolopoulos V. (2022). Global Impact of Delta plus Variant and Vaccination. Expert Rev. Vaccines.

[25] Classification of Omicron (B.1.1.529): SARS-CoV-2 Variant of Concern. https://www.who.int/news/item/26-11-2021-classification-of-omicron-(b.1.1.529)-sars-cov-2-variant-of-concern.

[26] Pulliam J.R., van Schalkwyk C., Govender N., von Gottberg A., Cohen C., Groome M.J., Dushoff J., Mlisana K., Moultrie H. (2021). Increased Risk of SARS-CoV-2 Reinfection Associated with Emergence of the Omicron Variant in South Africa. MedRxiv.

[27] Callaway E., Ledford H. (2021). How Bad Is Omicron? What Scientists Know so Far. Nature.

[28] Xiang F., Wang X., He X., Peng Z., Yang B., Zhang J., Zhou Q., Ye H., Ma Y., Li H. (2020). Antibody Detection and Dynamic Characteristics in Patients With Coronavirus Disease 2019. Clin. Infect. Dis..

[29] WHO (2021). Prevention SARS-CoV-2 Variant Classifications and Definitions. https://www.who.int/activities/tracking-SARS-CoV-2-variants.

[30] Preliminary Genomic Characterisation of an Emergent SARS-CoV-2 Lineage in the UK Defined by a Novel Set of Spike Mutations—SARS-CoV-2 Coronavirus/NCoV-2019 Genomic Epidemiology. https://virological.org/t/preliminary-genomic-characterisation-of-an-emergent-sars-cov-2-lineage-in-the-uk-defined-by-a-novel-set-of-spike-mutations/563.

[31] Horby P., Barclay W. (2021). NERVTAG Paper Brief Update on SARS-CoV-2 Variants [New Text in Red]. 5.

[32] Volz E., Mishra S., Chand M., Barrett J.C., Johnson R., Geidelberg L., Hinsley W.R., Laydon D.J., Dabrera G., The COVID-19 Genomics UK (COG-UK) consortium (2021). Assessing Transmissibility of SARS-CoV-2 Lineage B.1.1.7 in England. Nature.

[33] Krause P.R., Fleming T.R., Longini I.M., Peto R., Briand S., Heymann D.L., Beral V., Snape M.D., Rees H., Ropero A.-M. (2021). SARS-CoV-2 Variants and Vaccines. N. Engl. J. Med..

[34] Lauring A.S., Hodcroft E.B. (2021). Genetic Variants of SARS-CoV-2—What Do They Mean?. JAMA.

[35] Lorusso F., Inchingolo F., Scarano A. (2020). The Impact of COVID-19 on the Scientific Production Spread: A Five-Month Bibliometric Report of the Worldwide Research Community. Acta Med. Mediterr..

[36] Callaway E. (2020). The Coronavirus Is Mutating—Does It Matter?. Nature.

[37] Dao M.H., Phan L.T., Cao T.M., Luong Q.C., Pham H.T.T., Vu N.H.P., Khuu N.V., Nguyen T.V., Nguyen L.T., Nguyen H.T. (2021). Genome-wide Analysis of SARS-CoV-2 Strains Circulating in Vietnam: Understanding the Nature of the Epidemic and Role of the D614G Mutation. J. Med. Virol..

[38] Nguyen T.T., Pham T.N., Van T.D., Nguyen T.T., Nguyen D.T.N., Le H.N.M., Eden J.-S., Rockett R.J., Nguyen T.T.H., Vu B.T.N. (2020). Genetic Diversity of SARS-CoV-2 and Clinical, Epidemiological Characteristics of COVID-19 Patients in Hanoi, Vietnam. PLoS ONE.

[39] Aleem A., Akbar Samad A.B., Slenker A.K. (2021). Emerging Variants of SARS-CoV-2 And Novel Therapeutics Against Coronavirus (COVID-19). StatPearls.

[40] Kim Y.J., Jang U.S., Soh S.M., Lee J.-Y., Lee H.-R. (2021). The Impact on Infectivity and Neutralization Efficiency of SARS-CoV-2 Lineage B.1.351 Pseudovirus. Viruses.

[41] Yurkovetskiy L., Wang X., Pascal K.E., Tomkins-Tinch C., Nyalile T., Wang Y., Baum A., Diehl W.E., Dauphin A., Carbone C. (2020). Structural and Functional Analysis of the D614G SARS-CoV-2 Spike Protein Variant. Microbiology.

[42] Korber B., Fischer W.M., Gnanakaran S., Yoon H., Theiler J., Abfalterer W., Hengartner N., Giorgi E.E., Bhattacharya T., Foley B. (2020). Tracking Changes in SARS-CoV-2 Spike: Evidence That D614G Increases Infectivity of the COVID-19 Virus. Cell.

[43] Yurkovetskiy L., Pascal K.E., Tomkins-Tinch C., Nyalile T., Wang Y., Baum A., Diehl W.E., Dauphin A., Carbone C., Veinotte K. (2020). SARS-CoV-2 Spike Protein Variant D614G Increases Infectivity and Retains Sensitivity to Antibodies That Target the Receptor Binding Domain. BioRxiv.

[44] England P.H. (2021). SARS-CoV-2 Variants of Concern and Variants under Investigation in England. Public Health Engl..

[45] Yang Q., Meyerson N.R., Clark S.K., Paige C.L., Fattor W.T., Gilchrist A.R., Barbachano-Guerrero A., Healy B.G., Worden-Sapper E.R., Wu S.S. (2021). Saliva TwoStep for Rapid Detection of Asymptomatic SARS-CoV-2 Carriers. eLife.

[46] Sandoval Torrientes M., Castelló Abietar C., Boga Riveiro J., Álvarez-Argüelles M.E., Rojo-Alba S., Abreu Salinas F., Costales González I., Pérez Martínez Z., Martín Rodríguez G., Gómez de Oña J. (2021). A Novel Single Nucleotide Polymorphism Assay for the Detection of N501Y SARS-CoV-2 Variants. J. Virol. Methods.

[47] Vega-Magaña N., Sánchez-Sánchez R., Hernández-Bello J., Venancio-Landeros A.A., Peña-Rodríguez M., Vega-Zepeda R.A., Galindo-Ornelas B., Díaz-Sánchez M., García-Chagollán M., Macedo-Ojeda G. (2021). RT-QPCR Assays for Rapid Detection of the N501Y, 69-70del, K417N, and E484K SARS-CoV-2 Mutations: A Screening Strategy to Identify Variants with Clinical Impact. Front. Cell. Infect. Microbiol..

[48] Yaniv K., Ozer E., Shagan M., Lakkakula S., Plotkin N., Bhandarkar N.S., Kushmaro A. (2021). Direct RT-QPCR Assay for SARS-CoV-2 Variants of Concern (Alpha, B.1.1.7 and Beta, B.1.351) Detection and Quantification in Wastewater. Environ. Res..

[49] Vogels C.B.F., Breban M.I., Ott I.M., Alpert T., Petrone M.E., Watkins A.E., Kalinich C.C., Earnest R., Rothman J.E., Goes de Jesus J. (2021). Multiplex QPCR Discriminates Variants of Concern to Enhance Global Surveillance of SARS-CoV-2. PLoS Biol..

[50] La Rosa G., Mancini P., Bonanno Ferraro G., Veneri C., Iaconelli M., Lucentini L., Bonadonna L., Brusaferro S., Brandtner D., Fasanella A. (2021). Rapid Screening for SARS-CoV-2 Variants of Concern in Clinical and Environmental Samples Using Nested RT-PCR Assays Targeting Key Mutations of the Spike Protein. Water Res..

[51] Vircell https://en.vircell.com/.

[52] New VIASURE SARS-CoV-2 Variant I & Variant II RT-PCR Kits—CERTEST Biotec IVD Diagnostic Products. https://www.certest.es/news/new-viasure-sars-cov-2-variant-i-variant-ii-rt-pcr-kits/.

[53] Maglione M., Bevilacqua L., Dotto F., Costantinides F., Lorusso F., Scarano A. (2019). Observational Study on the Preparation of the Implant Site with Piezosurgery vs. Drill: Comparison between the Two Methods in Terms of Postoperative Pain, Surgical Times, and Operational Advantages. BioMed Res. Int..

[54] Inchingolo A.D., Dipalma G., Inchingolo A.M., Malcangi G., Santacroce L., D’oria M.T., Isacco C.G., Bordea I.R., Candrea S., Scarano A. (2021). The 15-Months Clinical Experience of SARS-CoV-2: A Literature Review of Therapies and Adjuvants. Antioxidants.

[55] Pham V.H., Gargiulo Isacco C., Nguyen K.C.D., Le S.H., Tran D.K., Nguyen Q.V., Pham H.T., Aityan S., Pham S.T., Cantore S. (2020). Rapid and Sensitive Diagnostic Procedure for Multiple Detection of Pandemic Coronaviridae Family Members SARS-CoV-2, SARS-CoV, MERS-CoV and HCoV: A Translational Research and Cooperation between the Phan Chau Trinh University in Vietnam and University of Bari “Aldo Moro” in Italy. Eur. Rev. Med. Pharm. Sci..

[56] GISAID—HCov19 Variants. https://www.gisaid.org/hcov19-variants/.

[57] Kimura I., Kosugi Y., Wu J., Yamasoba D., Butlertanaka E.P., Tanaka Y.L., Liu Y., Shirakawa K., Kazuma Y., Nomura R. (2021). SARS-CoV-2 Lambda Variant Exhibits Higher Infectivity and Immune Resistance. bioRxiv.

[58] Acevedo M.L., Alonso-Palomares L., Bustamante A., Gaggero A., Paredes F., Cortés C.P., Valiente-Echeverría F., Soto-Rifo R. (2021). Infectivity and Immune Escape of the New SARS-CoV-2 Variant of Interest Lambda. medRxiv.

[59] KFF COVID-19 Vaccine Breakthrough Cases: Data from the States. https://www.kff.org/policy-watch/covid-19-vaccine-breakthrough-cases-data-from-the-states/.

[60] Dhama K., Khan S., Tiwari R., Sircar S., Bhat S., Malik Y.S., Singh K.P., Chaicumpa W., Bonilla-Aldana D.K., Rodriguez-Morales A.J. (2020). Coronavirus Disease 2019–COVID-19. Clin. Microbiol. Rev..

[61] Christie A., Brooks J.T., Hicks L.A., Sauber-Schatz E.K., Yoder J.S., Honein M.A. (2021). CDC COVID-19 Response Team Guidance for Implementing COVID-19 Prevention Strategies in the Context of Varying Community Transmission Levels and Vaccination Coverage. MMWR Morb. Mortal. Wkly. Rep..

[62] Steensels D., Pierlet N., Penders J., Mesotten D., Heylen L. (2021). Comparison of SARS-CoV-2 Antibody Response Following Vaccination With BNT162b2 and MRNA-1273. JAMA.

[63] Latin America DW (2021). Coronavirus Lambda Variant Spreads across Latin America. https://www.dw.com/en/coronavirus-lambda-variant-spreads-across-latin-america/a-58035249.

[64] Nikolopoulou G.B., Maltezou H.C. (2021). COVID-19 in Children: Where Do We Stand?. Arch. Med. Res..

[65] Tipnis S.R., Hooper N.M., Hyde R., Karran E., Christie G., Turner A.J. (2000). A Human Homolog of Angiotensin-Converting Enzyme. J. Biol. Chem..

[66] Balzanelli M.G., Distratis P., Catucci O., Cefalo A., Lazzaro R., Inchingolo F., Tomassone D., Aityan S.K., Ballini A., Nguyen K.C. (2021). Mesenchymal Stem Cells: The Secret Children’s Weapons against the SARS-CoV-2 Lethal Infection. Appl. Sci..

[67] Dohan Ehrenfest D.M., de Peppo G.M., Doglioli P., Sammartino G. (2009). Slow Release of Growth Factors and Thrombospondin-1 in Choukroun’s Platelet-Rich Fibrin (PRF): A Gold Standard to Achieve for All Surgical Platelet Concentrates Technologies. Growth Factors.

[68] Cheng Y., Cheng G., Chui C.H., Lau F.Y., Chan P.K., Ng M.H., Sung J.J., Wong R.S. (2005). ABO Blood Group and Susceptibility to Severe Acute Respiratory Syndrome. JAMA.

[69] Breiman A., Ruvën-Clouet N., Le Pendu J. (2020). Harnessing the Natural Anti-Glycan Immune Response to Limit the Transmission of Enveloped Viruses Such as SARS-CoV-2. PLoS Pathog..

[70] Wu S.-C., Arthur C.M., Wang J., Verkerke H., Josephson C.D., Kalman D., Roback J.D., Cummings R.D., Stowell S.R. (2021). The SARS-CoV-2 Receptor-Binding Domain Preferentially Recognizes Blood Group A. Blood Adv..

[71] Cooling L. (2015). Blood Groups in Infection and Host Susceptibility. Clin. Microbiol. Rev..

[72] Anstee D.J. (2010). The Relationship between Blood Groups and Disease. Blood.

[73] Pillay T.S. (2020). Gene of the Month: The 2019-NCoV/SARS-CoV-2 Novel Coronavirus Spike Protein. J. Clin. Pathol..

[74] Jiang S., Du L., Shi Z. (2020). An Emerging Coronavirus Causing Pneumonia Outbreak in Wuhan, China: Calling for Developing Therapeutic and Prophylactic Strategies. Emerg. Microbes Infect..

[75] Bosch B.J., van der Zee R., de Haan C.A.M., Rottier P.J.M. (2003). The Coronavirus Spike Protein Is a Class I Virus Fusion Protein: Structural and Functional Characterization of the Fusion Core Complex. J. Virol..

[76] Inchingolo A.D., Inchingolo A.M., Bordea I.R., Malcangi G., Xhajanka E., Scarano A., Lorusso F., Farronato M., Tartaglia G.M., Isacco C.G. (2021). SARS-CoV-2 Disease through Viral Genomic and Receptor Implications: An Overview of Diagnostic and Immunology Breakthroughs. Microorganisms.

[77] Xia S., Zhu Y., Liu M., Lan Q., Xu W., Wu Y., Ying T., Liu S., Shi Z., Jiang S. (2020). Fusion Mechanism of 2019-NCoV and Fusion Inhibitors Targeting HR1 Domain in Spike Protein. Cell Mol. Immunol..

[78] Fung T.S., Liu D.X. (2018). Post-Translational Modifications of Coronavirus Proteins: Roles and Function. Future Virol..

[79] Zhu N., Zhang D., Wang W., Li X., Yang B., Song J., Zhao X., Huang B., Shi W., Lu R. (2020). A Novel Coronavirus from Patients with Pneumonia in China, 2019. N. Engl. J. Med..

[80] Zhou P., Yang X.-L., Wang X.-G., Hu B., Zhang L., Zhang W., Si H.-R., Zhu Y., Li B., Huang C.-L. (2020). A Pneumonia Outbreak Associated with a New Coronavirus of Probable Bat Origin. Nature.

[81] Zhang C., Zheng W., Huang X., Bell E.W., Zhou X., Zhang Y. (2020). Protein Structure and Sequence Reanalysis of 2019-NCoV Genome Refutes Snakes as Its Intermediate Host and the Unique Similarity between Its Spike Protein Insertions and HIV-1. J. Proteome Res..

[82] Zhang T., Wu Q., Zhang Z. (2020). Probable Pangolin Origin of SARS-CoV-2 Associated with the COVID-19 Outbreak. Curr. Biol..

[83] Guruprasad L. (2021). Human SARS-CoV-2 Spike Protein Mutations. Proteins: Struct. Funct. Bioinform..

[84] Wang Q., Zhang Y., Wu L., Niu S., Song C., Zhang Z., Lu G., Qiao C., Hu Y., Yuen K.-Y. (2020). Structural and Functional Basis of SARS-CoV-2 Entry by Using Human ACE2. Cell.

[85] Li F. (2016). Structure, Function, and Evolution of Coronavirus Spike Proteins. Annu. Rev. Virol..

[86] Weissenhorn W., Dessen A., Calder L.J., Harrison S.C., Skehel J.J., Wiley D.C. (1999). Structural Basis for Membrane Fusion by Enveloped Viruses. Mol. Membr. Biol..

[87] Balzanelli M.G., Distratis P., Lazzaro R., Cefalo A., Catucci O., Aityan S.K., Dipalma G., Vimercati L., Inchingolo A.D., Maggiore M.E. (2021). The Vitamin D, IL-6 and the EGFR Markers a Possible Way to Elucidate the Lung–Heart–Kidney Cross-Talk in COVID-19 Disease: A Foregone Conclusion. Microorganisms.

[88] Wrapp D., Wang N., Corbett K.S., Goldsmith J.A., Hsieh C.-L., Abiona O., Graham B.S., McLellan J.S. (2020). Cryo-EM Structure of the 2019-NCoV Spike in the Prefusion Conformation. Science.

[89] Hoffmann M., Kleine-Weber H., Schroeder S., Krüger N., Herrler T., Erichsen S., Schiergens T.S., Herrler G., Wu N.-H., Nitsche A. (2020). SARS-CoV-2 Cell Entry Depends on ACE2 and TMPRSS2 and Is Blocked by a Clinically Proven Protease Inhibitor. Cell.

[90] Tay M.Z., Poh C.M., Rénia L., MacAry P.A., Ng L.F.P. (2020). The Trinity of COVID-19: Immunity, Inflammation and Intervention. Nat Rev Immunol.

[91] Millet J.K., Whittaker G.R. (2015). Host Cell Proteases: Critical Determinants of Coronavirus Tropism and Pathogenesis. Virus Res..

[92] Walls A.C., Park Y.-J., Tortorici M.A., Wall A., McGuire A.T., Veesler D. (2020). Structure, Function, and Antigenicity of the SARS-CoV-2 Spike Glycoprotein. Cell.

[93] Matsuyama S., Nagata N., Shirato K., Kawase M., Takeda M., Taguchi F. (2010). Efficient Activation of the Severe Acute Respiratory Syndrome Coronavirus Spike Protein by the Transmembrane Protease TMPRSS2. J. Virol..

[94] Eckert D.M., Kim P.S. (2001). Mechanisms of Viral Membrane Fusion and Its Inhibition. Annu. Rev. Biochem..

[95] Masters P.S. (2006). The Molecular Biology of Coronaviruses. Advances in Virus Research.

[96] Hussain M., Jabeen N., Raza F., Shabbir S., Baig A.A., Amanullah A., Aziz B. (2020). Structural Variations in Human ACE2 May Influence Its Binding with SARS-CoV-2 Spike Protein. J. Med. Virol..

[97] Li W., Moore M.J., Vasilieva N., Sui J., Wong S.K., Berne M.A., Somasundaran M., Sullivan J.L., Luzuriaga K., Greenough T.C. (2003). Angiotensin-Converting Enzyme 2 Is a Functional Receptor for the SARS Coronavirus. Nature.

[98] Kuba K., Imai Y., Ohto-Nakanishi T., Penninger J.M. (2010). Trilogy of ACE2: A Peptidase in the Renin–Angiotensin System, a SARS Receptor, and a Partner for Amino Acid Transporters. Pharmacol. Ther..

[99] Verdecchia P., Cavallini C., Spanevello A., Angeli F. (2020). The Pivotal Link between ACE2 Deficiency and SARS-CoV-2 Infection. Eur. J. Intern. Med..

[100] Tetz G., Tetz V. (2020). SARS-CoV-2 Prion-Like Domains in Spike Proteins Enable Higher Affinity to ACE2. Microorganisms.

[101] Buzhdygan T.P., DeOre B.J., Baldwin-Leclair A., Bullock T.A., McGary H.M., Khan J.A., Razmpour R., Hale J.F., Galie P.A., Potula R. (2020). The SARS-CoV-2 Spike Protein Alters Barrier Function in 2D Static and 3D Microfluidic in-Vitro Models of the Human Blood–Brain Barrier. Neurobiol. Dis..

[102] Daneman R., Prat A. (2015). The Blood–Brain Barrier. Cold Spring Harb. Perspect. Biol..

[103] Daniels B.P., Holman D.W., Cruz-Orengo L., Jujjavarapu H., Durrant D.M., Klein R.S. (2014). Viral Pathogen-Associated Molecular Patterns Regulate Blood-Brain Barrier Integrity via Competing Innate Cytokine Signals. mBio.

[104] Grifoni A., Weiskopf D., Ramirez S.I., Mateus J., Dan J.M., Moderbacher C.R., Rawlings S.A., Sutherland A., Premkumar L., Jadi R.S. (2020). Targets of T Cell Responses to SARS-CoV-2 Coronavirus in Humans with COVID-19 Disease and Unexposed Individuals. Cell.

[105] Cao Y., Su B., Guo X., Sun W., Deng Y., Bao L., Zhu Q., Zhang X., Zheng Y., Geng C. (2020). Potent Neutralizing Antibodies against SARS-CoV-2 Identified by High-Throughput Single-Cell Sequencing of Convalescent Patients’ B Cells. Cell.

[106] Ju B., Zhang Q., Ge J., Wang R., Sun J., Ge X., Yu J., Shan S., Zhou B., Song S. (2020). Human Neutralizing Antibodies Elicited by SARS-CoV-2 Infection. Nature.

[107] Liu L., Wang P., Nair M.S., Yu J., Rapp M., Wang Q., Luo Y., Chan J.F.-W., Sahi V., Figueroa A. (2020). Potent Neutralizing Antibodies Directed to Multiple Epitopes on SARS-CoV-2 Spike. Microbiology.

[108] Brouwer P.J.M., Caniels T.G., van der Straten K., Snitselaar J.L., Aldon Y., Bangaru S., Torres J.L., Okba N.M.A., Claireaux M., Kerster G. (2020). Potent Neutralizing Antibodies from COVID-19 Patients Define Multiple Targets of Vulnerability. Science.

[109] Qin X., Shen J., Dai E., Li H., Tang G., Zhang L., Hou X., Lu M., Wu X., Duan S. (2021). The Seroprevalence and Kinetics of IgM and IgG in the Progression of COVID-19. BMC Immunol..

[110] Attolico I., Tarantini F., Carluccio P., Schifone C.P., Delia M., Gagliardi V.P., Perrone T., Gaudio F., Longo C., Giordano A. (2021). Serological Response Following BNT162b2 Anti-SARS-CoV-2 MRNA Vaccination in Haematopoietic Stem Cell Transplantation Patients. Br. J. Haematol..

[111] Qu J., Wu C., Li X., Zhang G., Jiang Z., Li X., Zhu Q., Liu L. (2020). Profile of Immunoglobulin G and IgM Antibodies Against Severe Acute Respiratory Syndrome Coronavirus 2 (SARS-CoV-2). Clin. Infect. Dis..

[112] Vimercati L., Stefanizzi P., De Maria L., Caputi A., Cavone D., Quarato M., Gesualdo L., Lopalco P.L., Migliore G., Sponselli S. (2021). Large-Scale IgM and IgG SARS-CoV-2 Serological Screening among Healthcare Workers with a Low Infection Prevalence Based on Nasopharyngeal Swab Tests in an Italian University Hospital: Perspectives for Public Health. Environ. Res.

[113] Nuccetelli M., Pieri M., Gisone F., Bernardini S. (2020). Combined Anti-SARS-CoV-2 IgA, IgG, and IgM Detection as a Better Strategy to Prevent Second Infection Spreading Waves. Immunol. Investig..

[114] Rhorer J., Ambrose C.S., Dickinson S., Hamilton H., Oleka N.A., Malinoski F.J., Wittes J. (2009). Efficacy of Live Attenuated Influenza Vaccine in Children: A Meta-Analysis of Nine Randomized Clinical Trials. Vaccine.

[115] Xu M., Wang D., Wang H., Zhang X., Liang T., Dai J., Li M., Zhang J., Zhang K., Xu D. (2020). COVID-19 Diagnostic Testing: Technology Perspective. Clin. Transl. Med..

[116] Kowitdamrong E., Puthanakit T., Jantarabenjakul W., Prompetchara E., Suchartlikitwong P., Putcharoen O., Hirankarn N. (2020). Antibody Responses to SARS-CoV-2 in Patients with Differing Severities of Coronavirus Disease 2019. PLoS ONE.

[117] Gao Z., Xu Y., Sun C., Wang X., Guo Y., Qiu S., Ma K. (2021). A Systematic Review of Asymptomatic Infections with COVID-19. J. Microbiol. Immunol. Infect..

[118] Fox A., Marino J., Amanat F., Krammer F., Hahn-Holbrook J., Zolla-Pazner S., Powell R.L. (2020). Robust and Specific Secretory IgA Against SARS-CoV-2 Detected in Human Milk. iScience.

[119] Mahmoodpoor A., Nader N.D. (2021). Immune Responses to the Novel Coronavirus-2: Friend or Foe?. Immunol. Investig..

[120] Agarwal A., Chen A., Ravindran N., To C., Thuluvath P.J. (2020). Gastrointestinal and Liver Manifestations of COVID-19. J. Clin. Exp. Hepatol..

[121] Ballini A., Dipalma G., Isacco C.G., Boccellino M., Di Domenico M., Santacroce L., Nguyễn K.C.D., Scacco S., Calvani M., Boddi A. (2020). Oral Microbiota and Immune System Crosstalk: A Translational Research. Biology.

[122] Guo L., Ren L., Yang S., Xiao M., Chang D., Yang F., Dela Cruz C.S., Wang Y., Wu C., Xiao Y. (2020). Profiling Early Humoral Response to Diagnose Novel Coronavirus Disease (COVID-19). Clin. Infect. Dis..

[123] Tetz G., Tetz V. (2022). Prion-Like Domains in Spike Protein of SARS-CoV-2 Differ across Its Variants and Enable Changes in Affinity to ACE2. Microorganisms.

[124] Ballini A., Santacroce L., Cantore S., Bottalico L., Dipalma G., Vito D.D., Saini R., Inchingolo F. (2018). Probiotics Improve Urogenital Health in Women. Open Access Maced. J. Med. Sci..

[125] Padoan A., Sciacovelli L., Basso D., Negrini D., Zuin S., Cosma C., Faggian D., Matricardi P., Plebani M. (2020). IgA-Ab Response to Spike Glycoprotein of SARS-CoV-2 in Patients with COVID-19: A Longitudinal Study. Clin. Chim. Acta.

[126] Padoan A., Cosma C., Sciacovelli L., Faggian D., Plebani M. (2020). Analytical Performances of a Chemiluminescence Immunoassay for SARS-CoV-2 IgM/IgG and Antibody Kinetics. Clin. Chem. Lab. Med. (CCLM).

[127] Chen L., Zhao J., Peng J., Li X., Deng X., Geng Z., Shen Z., Guo F., Zhang Q., Jin Y. (2020). Detection of SARS-CoV-2 in Saliva and Characterization of Oral Symptoms in COVID-19 Patients. Cell Prolif..

[128] Rapone B., Ferrara E., Santacroce L., Topi S., Gnoni A., Dipalma G., Mancini A., Di Domenico M., Tartaglia G.M., Scarano A. (2022). The Gaseous Ozone Therapy as a Promising Antiseptic Adjuvant of Periodontal Treatment: A Randomized Controlled Clinical Trial. Int. J. Environ. Res. Public Health.

[129] Phan D.Q., Nguyen L.D.N., Pham S.T., Nguyen T., Pham P.T.T., Nguyen S.T.H., Pham D.T., Pham H.T., Tran D.K., Le S.H. (2022). The Distribution of Dengue Virus Serotype in Quang Nam Province (Vietnam) during the Outbreak in 2018. Int. J. Environ. Res. Public Health.

[130] Indonesia First to Greenlight Novavax COVID-19 Vaccine | AP News. https://apnews.com/article/coronavirus-pandemic-technology-science-business-health-7e75808d4675e0e7597f52aeed1b8d5a.

[131] Algaissi A., Alfaleh M.A., Hala S., Abujamel T.S., Alamri S.S., Almahboub S.A., Alluhaybi K.A., Hobani H.I., Alsulaiman R.M., AlHarbi R.H. (2020). SARS-CoV-2 S1 and N-Based Serological Assays Reveal Rapid Seroconversion and Induction of Specific Antibody Response in COVID-19 Patients. Sci. Rep..

[132] Tang Y.-W., Schmitz J.E., Persing D.H., Stratton C.W. (2020). Laboratory Diagnosis of COVID-19: Current Issues and Challenges. J. Clin. Microbiol..

[133] Li Z., Yi Y., Luo X., Xiong N., Liu Y., Li S., Sun R., Wang Y., Hu B., Chen W. (2020). Development and Clinical Application of a Rapid IgM-IgG Combined Antibody Test for SARS-CoV-2 Infection Diagnosis. J. Med. Virol..

[134] Adeyinka A., Bailey K., Pierre L., Kondamudi N. (2021). COVID 19 Infection: Pediatric Perspectives. J. Am. Coll. Emerg. Physicians Open.

[135] Patel A., Jernigan D.B., Abdirizak F., Abedi G., Aggarwal S., Albina D., Allen E., Andersen L., Anderson J., Anderson M. (2020). Initial Public Health Response and Interim Clinical Guidance for the 2019 Novel Coronavirus Outbreak—United States, December 31, 2019–February 4, 2020. Morb. Mortal. Wkly. Rep..

[136] Bellocchio L., Inchingolo A.D., Inchingolo A.M., Lorusso F., Malcangi G., Santacroce L., Scarano A., Bordea I.R., Hazballa D., D’Oria M.T. (2021). Cannabinoids Drugs and Oral Health-From Recreational Side-Effects to Medicinal Purposes: A Systematic Review. Int. J. Mol. Sci..

[137] Taleghani N., Taghipour F. (2021). Diagnosis of COVID-19 for Controlling the Pandemic: A Review of the State-of-the-Art. Biosens. Bioelectron..

[138] Ejazi S.A., Ghosh S., Ali N. (2021). Antibody Detection Assays for COVID-19 Diagnosis: An Early Overview. Immunol. Cell Biol..

[139] Higgins V., Fabros A., Kulasingam V. (2021). Quantitative Measurement of Anti-SARS-CoV-2 Antibodies: Analytical and Clinical Evaluation. J. Clin. Microbiol..

[140] Rokni M., Ghasemi V., Tavakoli Z. (2020). Immune Responses and Pathogenesis of SARS-CoV-2 during an Outbreak in Iran: Comparison with SARS and MERS. Rev. Med. Virol..

[141] Tantuoyir M.M., Rezaei N. (2021). Serological Tests for COVID-19: Potential Opportunities. Cell Biol. Int..

[142] Werner M., Pervan P., Glück V., Zeman F., Koller M., Burkhardt R., Glück T., Wenzel J.J., Schmidt B., Gessner A. (2021). Evaluation of a Broad Panel of SARS-CoV-2 Serological Tests for Diagnostic Use. J. Clin. Med..

[143] Sun B., Feng Y., Mo X., Zheng P., Wang Q., Li P., Peng P., Liu X., Chen Z., Huang H. (2020). Kinetics of SARS-CoV-2 Specific IgM and IgG Responses in COVID-19 Patients. Emerg. Microbes Infect..

[144] Kyosei Y., Namba M., Makioka D., Kokubun A., Watabe S., Yoshimura T., Sasaki T., Shioda T., Ito E. (2021). Ultrasensitive Detection of SARS-CoV-2 Spike Proteins Using the Thio-NAD Cycling Reaction: A Preliminary Study before Clinical Trials. Microorganisms.

[145] Okba N.M., Müller M.A., Li W., Wang C., GeurtsvanKessel C.H., Corman V.M., Lamers M.M., Sikkema R.S., de Bruin E., Chandler F.D. (2020). Severe Acute Respiratory Syndrome Coronavirus 2- Specific Antibody Responses in Coronavirus Disease Patients. Emerg. Infect. Dis..

[146] Oguntuyo K.Y., Stevens C.S., Hung C.-T., Ikegame S., Acklin J.A., Kowdle S.S., Carmichael J.C., Chiu H.-P., Azarm K.D., Haas G.D. (2020). Quantifying Absolute Neutralization Titers against SARS-CoV-2 by a Standardized Virus Neutralization Assay Allows for Cross-Cohort Comparisons of COVID-19 Sera. Infect. Dis..

[147] Li G., Fan Y., Lai Y., Han T., Li Z., Zhou P., Pan P., Wang W., Hu D., Liu X. (2020). Coronavirus Infections and Immune Responses. J. Med. Virol..

[148] Sarvaria A., Madrigal J.A., Saudemont A. (2017). B Cell Regulation in Cancer and Anti-Tumor Immunity. Cell Mol. Immunol..

[149] Atri C., Guerfali F., Laouini D. (2018). Role of Human Macrophage Polarization in Inflammation during Infectious Diseases. IJMS.

[150] SeyedAlinaghi S., Mehrtak M., MohsseniPour M., Mirzapour P., Barzegary A., Habibi P., Moradmand-Badie B., Afsahi A.M., Karimi A., Heydari M. (2021). Genetic Susceptibility of COVID-19: A Systematic Review of Current Evidence. Eur. J. Med. Res..

[151] Mehta P., McAuley D.F., Brown M., Sanchez E., Tattersall R.S., Manson J.J. (2020). COVID-19: Consider Cytokine Storm Syndromes and Immunosuppression. Lancet.

[152] Sang E.R., Tian Y., Miller L.C., Sang Y. (2021). Epigenetic Evolution of ACE2 and IL-6 Genes: Non-Canonical Interferon-Stimulated Genes Correlate to COVID-19 Susceptibility in Vertebrates. Genes.

[153] Murakami M., Kamimura D., Hirano T. (2019). Pleiotropy and Specificity: Insights from the Interleukin 6 Family of Cytokines. Immunity.

[154] Garbers C., Heink S., Korn T., Rose-John S. (2018). Interleukin-6: Designing Specific Therapeutics for a Complex Cytokine. Nat. Rev. Drug Discov..

[155] Morelli C., Francavilla M., Stabile Ianora A.A., Cozzolino M., Gualano A., Stellacci G., Sacco A., Lorusso F., Pedote P., De Ceglie M. (2021). The Multifaceted COVID-19: CT Aspects of Its Atypical Pulmonary and Abdominal Manifestations and Complications in Adults and Children. A Pictorial Review. Microorganisms.

[156] Bhimraj A., Morgan R.L., Shumaker A.H., Lavergne V., Baden L., Cheng V.C.-C., Edwards K.M., Gandhi R., Muller W.J., O’Horo J.C. (2020). Infectious Diseases Society of America Guidelines on the Treatment and Management of Patients With Coronavirus Disease 2019 (COVID-19). Clin. Infect. Dis..

[157] Balzanelli G.M., Distratis P., Aityan S.K., Amatulli F., Catucci O., Cefalo A., Dipalma G., Inchingolo F., Lazzaro R., Nguyen K.C.D. (2020). COVID-19 and COVID-like Patients: A Brief Analysis and Findings of Two Deceased Cases. Open Access Maced. J. Med. Sci..

[158] Nguyen K.C. (2020). Could Immunity Boosting Therapy Help COVID-19 Patient?. BJSTR.

[159] Asano T., Boisson B., Onodi F., Matuozzo D., Moncada-Velez M., Maglorius Renkilaraj M.R.L., Zhang P., Meertens L., Bolze A., Materna M. (2021). X-Linked Recessive TLR7 Deficiency in ~1% of Men under 60 Years Old with Life-Threatening COVID-19. Sci. Immunol..

[160] Scarano A., Petrini M., Mastrangelo F., Noumbissi S., Lorusso F. (2020). The Effects of Liquid Disinfection and Heat Sterilization Processes on Implant Drill Roughness: Energy Dispersion X-Ray Microanalysis and Infrared Thermography. J. Clin. Med..

[161] Inchingolo A.D., Malcangi G., Inchingolo A.M., Piras F., Settanni V., Garofoli G., Palmieri G., Ceci S., Patano A., De Leonardis N. (2022). Benefits and Implications of Resveratrol Supplementation on Microbiota Modulations: A Systematic Review of the Literature. Int. J. Mol. Sci..

[162] Zhang Q., Bastard P., Liu Z., Le Pen J., Moncada-Velez M., Chen J., Ogishi M., Sabli I.K.D., Hodeib S., Korol C. (2020). Inborn Errors of Type I IFN Immunity in Patients with Life-Threatening COVID-19. Science.

[163] Balzanelli G.M., Distratis P., Amatulli F., Catucci O., Cefalo A., Lazzaro R., Palazzo D., Aityan K.S., Dipalma G., Inchingolo F. (2020). Clinical Features in Predicting COVID-19. Biomed. J. Sci. Tech. Res..

[164] Balzanelli M.G., Distratis P., Lazzaro R., Cefalo A., Dangela G., Catucci O., Palazzo D., Amatulli F., Tomassone D., Pham V. (2020). Would the End Of COVID-19 Infection as a Chronic Disease?. J. Stem Cells Res. Dev. Ther..

[165] Balzanelli M.G., Distratis P., Aityan S.K., Amatulli F., Catucci O., Cefalo A., De Michele A., Dipalma G., Inchingolo F., Lazzaro R. (2021). An Alternative “Trojan Horse” Hypothesis for COVID-19: Immune Deficiency of IL-10 and SARS-CoV-2 Biology. EMIDDT.

[166] Bevilacqua V., Altini N., Prencipe B., Brunetti A., Villani L., Sacco A., Morelli C., Ciaccia M., Scardapane A. (2021). Lung Segmentation and Characterization in COVID-19 Patients for Assessing Pulmonary Thromboembolism: An Approach Based on Deep Learning and Radiomics. Electronics.

[167] Bavaro D.F., Poliseno M., Scardapane A., Belati A., De Gennaro N., Stabile Ianora A.A., Angarano G., Saracino A. (2020). Occurrence of Acute Pulmonary Embolism in COVID-19-A Case Series. Int. J. Infect. Dis..

[168] Scardapane A., Villani L., Bavaro D.F., Passerini F., Ianora A.A.S., Lucarelli N.M., Angarano G., Portincasa P., Palmieri V.O., Saracino A. (2021). Pulmonary Artery Filling Defects in COVID-19 Patients Revealed Using CT Pulmonary Angiography: A Predictable Complication?. Biomed. Res. Int..

[169] Spencer J.P., Trondsen Pawlowski R.H., Thomas S. (2017). Vaccine Adverse Events: Separating Myth from Reality. Am. Fam. Physician.

[170] Krammer F. (2020). SARS-CoV-2 Vaccines in Development. Nature.

[171] U.S. Food and Drug Administration FDA Approves First COVID-19 Vaccine. https://www.fda.gov/news-events/press-announcements/fda-approves-first-covid-19-vaccine.

[172] EMA First COVID-19 Vaccine Approved for Children Aged 12 to 15 in EU. https://www.ema.europa.eu/en/news/first-covid-19-vaccine-approved-children-aged-12-15-eu.

[173] Dai L., Gao G.F. (2021). Viral Targets for Vaccines against COVID-19. Nat. Rev. Immunol..

[174] Gobbi F., Buonfrate D., Moro L., Rodari P., Piubelli C., Caldrer S., Riccetti S., Sinigaglia A., Barzon L. (2021). Antibody Response to the BNT162b2 MRNA COVID-19 Vaccine in Subjects with Prior SARS-CoV-2 Infection. Viruses.

[175] Bianchi F.P., Germinario C.A., Migliore G., Vimercati L., Martinelli A., Lobifaro A., Tafuri S., Stefanizzi P., Control Room Working Group (2021). BNT162b2 MRNA COVID-19 Vaccine Effectiveness in the Prevention of SARS-CoV-2 Infection: A Preliminary Report. J. Infect. Dis..

[176] Greenwood B. (2014). The Contribution of Vaccination to Global Health: Past, Present and Future. Philos. Trans. R. Soc. B Biol. Sci..

[177] Lurie N., Saville M., Hatchett R., Halton J. (2020). Developing COVID-19 Vaccines at Pandemic Speed. N. Engl. J. Med..

[178] Chung J.Y., Thone M.N., Kwon Y.J. (2021). COVID-19 Vaccines: The Status and Perspectives in Delivery Points of View. Adv. Drug Deliv. Rev..

[179] Chakraborty S., Mallajosyula V., Tato C.M., Tan G.S., Wang T.T. (2021). SARS-CoV-2 Vaccines in Advanced Clinical Trials: Where Do We Stand?. Adv. Drug Deliv. Rev..

[180] Wang H., Zhang Y., Huang B., Deng W., Quan Y., Wang W., Xu W., Zhao Y., Li N., Zhang J. (2020). Development of an Inactivated Vaccine Candidate, BBIBP-CorV, with Potent Protection against SARS-CoV-2. Cell.

[181] Xia S., Duan K., Zhang Y., Zhao D., Zhang H., Xie Z., Li X., Peng C., Zhang Y., Zhang W. (2020). Effect of an Inactivated Vaccine against SARS-CoV-2 on Safety and Immunogenicity Outcomes: Interim Analysis of 2 Randomized Clinical Trials. JAMA.

[182] Gao Q., Bao L., Mao H., Wang L., Xu K., Yang M., Li Y., Zhu L., Wang N., Lv Z. (2020). Development of an Inactivated Vaccine Candidate for SARS-CoV-2. Science.

[183] Palacios R., Patiño E.G., de Oliveira Piorelli R., Conde M.T.R.P., Batista A.P., Zeng G., Xin Q., Kallas E.G., Flores J., Ockenhouse C.F. (2020). Double-Blind, Randomized, Placebo-Controlled Phase III Clinical Trial to Evaluate the Efficacy and Safety of Treating Healthcare Professionals with the Adsorbed COVID-19 (Inactivated) Vaccine Manufactured by Sinovac—PROFISCOV: A Structured Summary of a Study Protocol for a Randomised Controlled Trial. Trials.

[184] Mallapaty S. (2021). China COVID Vaccine Reports Mixed Results—What Does That Mean for the Pandemic?. Nature.

[185] Prevention B.V. (2020). A Randomized, Double-Blind, Placebo-Controlled Phase 3 Study to Assess the Efficacy and Safety of Ad26. COV2. S for the Prevention of SARS-CoV-2-Mediated COVID-19 in Adults Aged 18 Years and Older.

[186] Sheikh A.B., Pal S., Javed N., Shekhar R. (2021). COVID-19 Vaccination in Developing Nations: Challenges and Opportunities for Innovation. Infect. Dis. Rep..

[187] Logunov D.Y., Dolzhikova I.V., Shcheblyakov D.V., Tukhvatulin A.I., Zubkova O.V., Dzharullaeva A.S., Kovyrshina A.V., Lubenets N.L., Grousova D.M., Erokhova A.S. (2021). Safety and Efficacy of an RAd26 and RAd5 Vector-Based Heterologous Prime-Boost COVID-19 Vaccine: An Interim Analysis of a Randomised Controlled Phase 3 Trial in Russia. Lancet.

[188] Bangaru S., Ozorowski G., Turner H.L., Antanasijevic A., Huang D., Wang X., Torres J.L., Diedrich J.K., Tian J.-H., Portnoff A.D. (2020). Structural Analysis of Full-Length SARS-CoV-2 Spike Protein from an Advanced Vaccine Candidate. Science.

[189] BioNTech SE A PHASE 1/2/3, Placebo-Controlled, Randomized, Observer-Blind, Dose-Finding Study to Evaluate the Safety, Tolerability, Immunogenicity, and Efficacy of SARS-CoV-2 RNA Vaccine Candidates against COVID-19 in Healthy Indi-viduals. clinicaltrials.gov.

[190] Mahase E. (2020). COVID-19: Moderna Vaccine Is Nearly 95% Effective, Trial Involving High Risk and Elderly People Shows. BMJ Br. Med. J. (Online).

[191] Mahase E. (2020). COVID-19: UK Government Asks Regulator to Assess Oxford Vaccine as Questions Are Raised over Interim Data. BMJ Br. Med. J. (Online).

[192] EMA EMA and ECDC Update on COVID-19. https://www.ema.europa.eu/en/news/ema-ecdc-update-covid-19.

[193] National Cohort Study of Effectiveness and Safety of SARS-CoV-2/COVID-19 Vaccines. clinicaltrials.gov.

[194] Fabiani M., Ramigni M., Gobbetto V., Mateo-Urdiales A., Pezzotti P., Piovesan C. (2021). Effectiveness of the Comirnaty (BNT162b2, BioNTech/Pfizer) Vaccine in Preventing SARS-CoV-2 Infection among Healthcare Workers, Treviso Province, Veneto Region, Italy, 27 December 2020 to 24 March 2021. Eurosurveillance.

[195] BioNTech/Pfizer Vaccine Authorised Answers to Frequently Asked Questions on AIFA’s Website. https://www.aifa.gov.it/en/-/autorizzato-il-vaccino-biontech-pfizer.

[196] Abbasi J. (2020). COVID-19 and MRNA Vaccines—First Large Test for a New Approach. JAMA.

[197] https://www.ema.europa.eu/en/medicines/human/epar/comirnaty.

[198] Development of Therapeutics and Vaccines. https://www.eu-patient.eu/COVID-19/covid-resource-point/therapeutics-and-vaccines/.

[199] Hou X., Zaks T., Langer R., Dong Y. (2021). Lipid Nanoparticles for MRNA Delivery. Nat. Rev. Mater..

[200] Lamb Y.N. (2021). BNT162b2 MRNA COVID-19 Vaccine: First Approval. Drugs.

[201] Frenck R.W., Klein N.P., Kitchin N., Gurtman A., Absalon J., Lockhart S., Perez J.L., Walter E.B., Senders S., Bailey R. (2021). Safety, Immunogenicity, and Efficacy of the BNT162b2 COVID-19 Vaccine in Adolescents. N. Engl. J. Med..

[202] Sahin U., Muik A., Vogler I., Derhovanessian E., Kranz L.M., Vormehr M., Quandt J., Bidmon N., Ulges A., Baum A. (2020). BNT162b2 Induces SARS-CoV-2-Neutralising Antibodies and T Cells in Humans; Infectious Diseases (except HIV/AIDS). medRxiv.

[203] Dagan N., Barda N., Kepten E., Miron O., Perchik S., Katz M.A., Hernán M.A., Lipsitch M., Reis B., Balicer R.D. (2021). BNT162b2 MRNA COVID-19 Vaccine in a Nationwide Mass Vaccination Setting. N. Engl. J. Med..

[204] Thompson M.G., Burgess J.L., Naleway A.L., Tyner H.L., Yoon S.K., Meece J., Olsho L.E.W., Caban-Martinez A.J., Fowlkes A., Lutrick K. (2021). Interim Estimates of Vaccine Effectiveness of BNT162b2 and MRNA-1273 COVID-19 Vaccines in Preventing SARS-CoV-2 Infection Among Health Care Personnel, First Responders, and Other Essential and Frontline Workers—Eight U.S. Locations, December 2020–March 2021. MMWR Morb. Mortal. Wkly. Rep..

[205] Patano A., Cirulli N., Beretta M., Plantamura P., Inchingolo A.D., Inchingolo A.M., Bordea I.R., Malcangi G., Marinelli G., Scarano A. (2021). Education Technology in Orthodontics and Paediatric Dentistry during the COVID-19 Pandemic: A Systematic Review. Int. J. Environ. Res. Public Health.

[206] Bordea I.R., Xhajanka E., Candrea S., Bran S., Onișor F., Inchingolo A.D., Malcangi G., Pham V.H., Inchingolo A.M., Scarano A. (2020). Coronavirus (SARS-CoV-2) Pandemic: Future Challenges for Dental Practitioners. Microorganisms.

[207] Swann O.V., Holden K.A., Turtle L., Pollock L., Fairfield C.J., Drake T.M., Seth S., Egan C., Hardwick H.E., Halpin S. (2020). Clinical Characteristics of Children and Young People Admitted to Hospital with COVID-19 in United Kingdom: Prospective Multicentre Observational Cohort Study. BMJ.

[208] Dohan Ehrenfest D.M., Del Corso M., Inchingolo F., Sammartino G., Charrier J.-B. (2010). Platelet-Rich Plasma (PRP) and Platelet-Rich Fibrin (PRF) in Human Cell Cultures: Growth Factor Release and Contradictory Results. Oral Surg. Oral Med. Oral Pathol. Oral Radiol. Endod..

[209] King H., Deshpande S., Woodbridge T., Hilliard T., Standing J., Lewis M., Ward L., Finn A., Roderick M. (2022). Initial Experience of the Safety and Tolerability of the BNT162b2 (Pfizer-Bio-N-Tech) Vaccine in Extremely Vulnerable Children Aged 12–15 Years. Arch. Dis. Child..

[210] Callaway E. (2020). What Pfizer’s Landmark COVID Vaccine Results Mean for the Pandemic. Nature.

[211] WHO (2021). Vaccine Tracker and Landscape.

[212] Draft Landscape of COVID-19 Candidate Vaccines. https://www.who.int/publications/m/item/draft-landscape-of-covid-19-candidate-vaccines.

[213] Mulligan M.J., Lyke K.E., Kitchin N., Absalon J., Gurtman A., Lockhart S., Neuzil K., Raabe V., Bailey R., Swanson K.A. (2020). Phase I/II Study of COVID-19 RNA Vaccine BNT162b1 in Adults. Nature.

[214] Pfizer U.S. CDC Committee of Independent Health Experts Recommends Vaccination with Pfizer and Biontech COVID-19 Vaccine for Persons Ages 16 Years and Older 2020. https://www.pfizer.com/news/press-release/press-release-detail/us-cdc-committee-independent-health-experts-recommends.

[215] Liu Y., Liu J., Xia H., Zhang X., Fontes-Garfias C.R., Swanson K.A., Cai H., Sarkar R., Chen W., Cutler M. (2021). Neutralizing Activity of BNT162b2-Elicited Serum. N. Engl. J. Med..

[216] Barda N., Dagan N., Ben-Shlomo Y., Kepten E., Waxman J., Ohana R., Hernán M.A., Lipsitch M., Kohane I., Netzer D. (2021). Safety of the BNT162b2 MRNA COVID-19 Vaccine in a Nationwide Setting. N. Engl. J. Med..

[217] Oliver S.E., Gargano J.W., Marin M., Wallace M., Curran K.G., Chamberland M., McClung N., Campos-Outcalt D., Morgan R.L., Mbaeyi S. (2021). The Advisory Committee on Immunization Practices’ Interim Recommendation for Use of Moderna COVID-19 Vaccine—United States, December 2020. MMWR Morb. Mortal. Wkly. Rep..

[218] COVID-19 Vaccine Tracker and Landscape. https://www.who.int/publications/m/item/draft-landscape-of-covid-19-candidate-vaccines.

[219] Fix O.K., Blumberg E.A., Chang K., Chu J., Chung R.T., Goacher E.K., Hameed B., Kaul D.R., Kulik L.M., Kwok R.M. (2021). American Association for the Study of Liver Diseases Expert Panel Consensus Statement: Vaccines to Prevent Coronavirus Disease 2019 Infection in Patients With Liver Disease. Hepatology.

[220] COVID-19, Il Vaccino Astra Zeneca Efficace “per Errore” Univadis. https://www.univadis.it/viewarticle/covid-19-il-vaccino-astra-zeneca-efficace-per-errore-734126.

[221] Emary K.R.W., Golubchik T., Aley P.K., Ariani C.V., Angus B., Bibi S., Blane B., Bonsall D., Cicconi P., Charlton S. (2021). Efficacy of ChAdOx1 NCoV-19 (AZD1222) Vaccine against SARS-CoV-2 Variant of Concern 202012/01 (B.1.1.7): An Exploratory Analysis of a Randomised Controlled Trial. Lancet.

[222] PINHO A.C. COVID-19 Vaccine AstraZeneca: PRAC Preliminary View Suggests No Specific Issue with Batch Used in Austria. https://www.ema.europa.eu/en/news/covid-19-vaccine-astrazeneca-prac-preliminary-view-suggests-no-specific-issue-batch-used-austria.

[223] AIFA Imposes Ban of Use of AstraZeneca Batch. Investigations in Progress in Coordination with EMA. https://www.aifa.gov.it/en/web/guest/-/aifa-dispone-divieto-di-utilizzo-di-un-lotto-astrazeneca-accertamenti-in-corso-in-coordinamento-con-ema.

[224] Bertelli M., Kiani A.K., Paolacci S., Manara E., Kurti D., Dhuli K., Bushati V., Miertus J., Pangallo D., Baglivo M. (2020). Hydroxytyrosol: A Natural Compound with Promising Pharmacological Activities. J. Biotechnol..

[225] Ergoren M.C., Paolacci S., Manara E., Dautaj A., Dhuli K., Anpilogov K., Camilleri G., Suer H.K., Sayan M., Tuncel G. (2020). A Pilot Study on the Preventative Potential of Alpha-Cyclodextrin and Hydroxytyrosol against SARS-CoV-2 Transmission. Acta Bio Med. Atenei Parm..

[226] Burgos R.M., Badowski M.E., Drwiega E., Ghassemi S., Griffith N., Herald F., Johnson M., Smith R.O., Michienzi S.M. (2021). The Race to a COVID-19 Vaccine: Opportunities and Challenges in Development and Distribution. DIC.

[227] Haynes B.F., Corey L., Fernandes P., Gilbert P.B., Hotez P.J., Rao S., Santos M.R., Schuitemaker H., Watson M., Arvin A. (2020). Prospects for a Safe COVID-19 Vaccine. Sci. Transl. Med..

[228] Commissioner O. of the FDA Issues Emergency Use Authorization for Third COVID-19 Vaccine. https://www.fda.gov/news-events/press-announcements/fda-issues-emergency-use-authorization-third-covid-19-vaccine.

[229] Statement from NIH and BARDA on the FDA Emergency Use Authorization of the Janssen COVID-19 Vaccine|National Institutes of Health (NIH). https://www.nih.gov/news-events/news-releases/statement-nih-barda-fda-emergency-use-authorization-janssen-covid-19-vaccine.

[230] Ashraf M.U., Kim Y., Kumar S., Seo D., Ashraf M., Bae Y.-S. (2021). COVID-19 Vaccines (Revisited) and Oral-Mucosal Vector System as a Potential Vaccine Platform. Vaccines.

[231] Zhang H., Kang Z., Gong H., Xu D., Wang J., Li Z., Li Z., Cui X., Xiao J., Zhan J. (2020). Digestive System Is a Potential Route of COVID-19: An Analysis of Single-Cell Coexpression Pattern of Key Proteins in Viral Entry Process. Gut.

[232] Hou Y.J., Okuda K., Edwards C.E., Martinez D.R., Asakura T., Dinnon K.H., Kato T., Lee R.E., Yount B.L., Mascenik T.M. (2020). SARS-CoV-2 Reverse Genetics Reveals a Variable Infection Gradient in the Respiratory Tract. Cell.

[233] Sims A.C., Baric R.S., Yount B., Burkett S.E., Collins P.L., Pickles R.J. (2005). Severe Acute Respiratory Syndrome Coronavirus Infection of Human Ciliated Airway Epithelia: Role of Ciliated Cells in Viral Spread in the Conducting Airways of the Lungs. J. Virol..

[234] Biopharma-Reporter.Com Vaxart’s Oral COVID-19 Tablet V—Cerca Con Google. https://www.google.it/search?q=biopharma-reporter.com+Vaxart%E2%80%99s+Oral+COVID-19+Tablet+V&ei=ribNYuvfOK2-xc8PsdSioAo&ved=0ahUKEwjr7orc7fL4AhUtX_EDHTGqCKQQ4dUDCA4&uact=5&oq=biopharma-reporter.com+Vaxart%E2%80%99s+Oral+COVID-19+Tablet+V&gs_lcp=Cgdnd3Mtd2l6EAMyBAghEBVKBAhBGAFKBAhGGABQzgdYzgdglgtoAXAAeACAAbcCiAG3ApIBAzMtMZgBAKABAqABAcABAQ&sclient=gws-wiz.

[235] Iwasaki A., Pillai P.S. (2014). Innate Immunity to Influenza Virus Infection. Nat. Rev. Immunol..

[236] iosBio Stabilitech Biopharma Announces Name Change to IosBio. https://www.globenewswire.com/news-release/2020/09/30/2101099/0/en/Stabilitech-Biopharma-announces-name-change-to-iosBio.html.

[237] Callaway E. (2021). COVID Vaccines and Kids: Five Questions as Trials Begin. Nature.

[238] Montenegro V., Inchingolo A.D., Malcangi G., Limongelli L., Marinelli G., Coloccia G., Laudadio C., Patano A., Inchingolo F., Bordea I.R. (2021). Compliance of Children with Removable Functional Appliance with Microchip Integrated during COVID-19 Pandemic: A Systematic Review. J. Biol. Regul. Homeost. Agents.

[239] Rose A., Stevo C., Alatovic J., Maas S. (2021). Pfizer and BioNTech Announce Positive Topline Results from Pivotal Trial of COVID-19 Vaccine in Children 5 to 11 Years.

[240] (2021). 240. Reuters CDC Backs Pfizer COVID-19 Vaccine for Young Children. The Guardian.

[241] CDC COVID-19 Vaccines for Children & Teens. https://www.cdc.gov/coronavirus/2019-ncov/vaccines/recommendations/children-teens.html.

[242] Baang J.H., Smith C., Mirabelli C., Valesano A.L., Manthei D.M., Bachman M.A., Wobus C.E., Adams M., Washer L., Martin E.T. (2021). Prolonged Severe Acute Respiratory Syndrome Coronavirus 2 Replication in an Immunocompromised Patient. J. Infect. Dis..

[243] Mullins E., Hudak M.L., Banerjee J., Getzlaff T., Townson J., Barnette K., Playle R., Perry A., Bourne T., Lees C.C. (2021). Pregnancy and Neonatal Outcomes of COVID-19: Coreporting of Common Outcomes from PAN-COVID and AAP-SONPM Registries. Ultrasound Obstet. Gynecol..

[244] Kadiwar S., Smith J.J., Ledot S., Johnson M., Bianchi P., Singh N., Montanaro C., Gatzoulis M., Shah N., Ukor E.-F. (2021). Were Pregnant Women More Affected by COVID-19 in the Second Wave of the Pandemic?. Lancet.

[245] Malcangi G., Inchingolo A.D., Inchingolo A.M., Santacroce L., Marinelli G., Mancini A., Vimercati L., Maggiore M.E., D’Oria M.T., Hazballa D. (2021). COVID-19 Infection in Children, Infants and Pregnant Subjects: An Overview of Recent Insights and Therapies. Microorganisms.

[246] Riley L.E., Jamieson D.J. (2021). Inclusion of Pregnant and Lactating Persons in COVID-19 Vaccination Efforts. Ann. Intern. Med..

[247] Mangat C., Milosavljevic N. (2021). BNT162b2 Vaccination during Pregnancy Protects Both the Mother and Infant: Anti-SARS-CoV-2 S Antibodies Persistently Positive in an Infant at 6 Months of Age. Case Rep. Pediatr..

[248] Blumberg D., Sridhar A., Lakshminrusimha S., Higgins R.D., Saade G. (2021). COVID-19 Vaccine Considerations during Pregnancy and Lactation. Am. J. Perinatol..

[249] Pace R.M., Williams J.E., Järvinen K.M., Belfort M.B., Pace C.D., Lackey K.A., Gogel A.C., Nguyen-Contant P., Kanagaiah P., Fitzgerald T. (2020). COVID-19 and Human Milk: SARS-CoV-2, Antibodies, and Neutralizing Capacity. medRxiv.

[250] Aygun H. (2020). Vitamin D Can Prevent COVID-19 Infection-Induced Multiple Organ Damage. Naunyn Schmiedebergs Arch. Pharm..

[251] Science M., Maguire J.L., Russell M.L., Smieja M., Walter S.D., Loeb M. (2013). Low Serum 25-Hydroxyvitamin D Level and Risk of Upper Respiratory Tract Infection in Children and Adolescents. Clin. Infect. Dis..

[252] Gunasekar P., Swier V.J., Fleegel J.P., Boosani C.S., Radwan M.M., Agrawal D.K. (2018). Vitamin D and Macrophage Polarization in Epicardial Adipose Tissue of Atherosclerotic Swine. PLoS ONE.

[253] Charitos I.A., Ballini A., Bottalico L., Cantore S., Passarelli P.C., Inchingolo F., D’Addona A., Santacroce L. (2020). Special Features of SARS-CoV-2 in Daily Practice. WJCC.

[254] Vimercati L., De Maria L., Quarato M., Caputi A., Gesualdo L., Migliore G., Cavone D., Sponselli S., Pipoli A., Inchingolo F. (2021). Association between Long COVID and Overweight/Obesity. JCM.

[255] Santacroce L., Charitos I.A., Ballini A., Inchingolo F., Luperto P., De Nitto E., Topi S. (2020). The Human Respiratory System and Its Microbiome at a Glimpse. Biology.

[256] Wang Z., Schmidt F., Weisblum Y., Muecksch F., Barnes C.O., Finkin S., Schaefer-Babajew D., Cipolla M., Gaebler C., Lieberman J.A. (2021). MRNA Vaccine-Elicited Antibodies to SARS-CoV-2 and Circulating Variants. Immunology.

[257] Ai-ris Y.C., McMahan K., Yu J., Tostanoski L.H., Aguayo R., Ansel J., Chandrashekar A., Patel S., Bondzie E.A., Sellers D. (2021). Immunogenicity of COVID-19 MRNA Vaccines in Pregnant and Lactating Women. JAMA.

[258] Zeng H., Xu C., Fan J., Tang Y., Deng Q., Zhang W., Long X. (2020). Antibodies in Infants Born to Mothers With COVID-19 Pneumonia. JAMA.

[259] Kohler P.F., Farr R.S. (1966). Elevation of Cord over Maternal IgG Immunoglobulin: Evidence for an Active Placental IgG Transport. Nature.

[260] Ng W.F., Wong S.F., Lam A., Mak Y.F., Yao H., Lee K.C., Chow K.M., Yu W.C., Ho L.C. (2006). The Placentas of Patients with Severe Acute Respiratory Syndrome: A Pathophysiological Evaluation. Pathology.

[261] Perl S.H., Uzan-Yulzari A., Klainer H., Asiskovich L., Youngster M., Rinott E., Youngster I. (2021). SARS-CoV-2–Specific Antibodies in Breast Milk after COVID-19 Vaccination of Breastfeeding Women. JAMA.

[262] Berry S.D., Johnson K.S., Myles L., Herndon L., Montoya A., Fashaw S., Gifford D. (2021). Lessons Learned from Frontline Skilled Nursing Facility Staff Regarding COVID-19 Vaccine Hesitancy. J. Am. Geriatr. Soc..

[263] Gonzalez D.C., Nassau D.E., Khodamoradi K., Ibrahim E., Blachman-Braun R., Ory J., Ramasamy R. (2021). Sperm Parameters Before and After COVID-19 MRNA Vaccination. JAMA.

[264] Al-Ansari R.Y., Al-Sharari M., Al-Saadi T. (2021). Palms and Soles Itchiness as a Side Effect of COVID-19 Vaccination. J. Infect Public Health.

[265] Larson K.F., Ammirati E., Adler E.D., Cooper L.T., Hong K.N., Saponara G., Couri D., Cereda A., Procopio A., Cavalotti C. (2021). Myocarditis After BNT162b2 and MRNA-1273 Vaccination. Circulation.

[266] Bozkurt B., Kamat I., Hotez P.J. (2021). Myocarditis With COVID-19 MRNA Vaccines. Circulation.

[267] Marshall M., Ferguson I.D., Lewis P., Jaggi P., Gagliardo C., Collins J.S., Shaughnessy R., Caron R., Fuss C., Corbin K.J.E. (2021). Symptomatic Acute Myocarditis in 7 Adolescents after Pfizer-BioNTech COVID-19 Vaccination. Pediatrics.

[268] Lu L., Xiong W., Mu J., Zhang Q., Zhang H., Zou L., Li W., He L., Sander J.W., Zhou D. (2021). The Potential Neurological Effect of the COVID-19 Vaccines: A Review. Acta Neurol. Scand..

[269] Zhao H., Souders C., Carmel M., Anger J.T. (2021). Low Rates of Urologic Side Effects Following Coronavirus Disease Vaccination: An Analysis of the Food and Drug Administration Vaccine Adverse Event Reporting System. Urology.

[270] Arora P., Sardana K., Mathachan S.R., Malhotra P. (2021). Herpes Zoster after Inactivated COVID-19 Vaccine: A Cutaneous Adverse Effect of the Vaccine. J Cosmet Dermtol..

[271] Kayser F., Fourneau H., Mazy O.C., Mazy S. (2021). Breast Implant Seroma: A SARS-CoV-2 MRNA Vaccine Side Effect. J. Clin. Ultrasound..

[272] Esba L.C.A., Al Jeraisy M. (2021). Reported Adverse Effects Following COVID-19 Vaccination at a Tertiary Care Hospital, Focus on Cerebral Venous Sinus Thrombosis (CVST). Expert Rev. Vaccines.

[273] Lebedev L., Sapojnikov M., Wechsler A., Varadi-Levi R., Zamir D., Tobar A., Levin-Iaina N., Fytlovich S., Yagil Y. (2021). Minimal Change Disease Following the Pfizer-BioNTech COVID-19 Vaccine. Am. J. Kidney Dis..

[274] Fowler N., Mendez Martinez N.R., Pallares B.V., Maldonado R.S. (2021). Acute-Onset Central Serous Retinopathy after Immunization with COVID-19 MRNA Vaccine. Am. J. Ophthalmol. Case Rep..

[275] Mahajan S., Zhang F., Mahajan A., Zimnowodzki S. (2021). Parsonage Turner Syndrome after COVID-19 Vaccination. Muscle Nerve.

[276] Cirillo N. (2021). Reported Orofacial Adverse Effects of COVID-19 Vaccines: The Knowns and the Unknowns. J. Oral Pathol. Med..

[277] Grupper A., Rabinowich L., Schwartz D., Schwartz I.F., Ben-Yehoyada M., Shashar M., Katchman E., Halperin T., Turner D., Goykhman Y. (2021). Reduced Humoral Response to MRNA SARS-CoV-2 BNT162b2 Vaccine in Kidney Transplant Recipients without Prior Exposure to the Virus. Am. J. Transpl..

[278] Mazzola A., Todesco E., Drouin S., Hazan F., Marot S., Thabut D., Varnous S., Soulié C., Barrou B., Marcelin A.-G. (2022). Poor Antibody Response After Two Doses of Severe Acute Respiratory Syndrome Coronavirus 2 (SARS-CoV-2) Vaccine in Transplant Recipients. Clin. Infect. Dis..

[279] Hause A.M., Gee J., Baggs J., Abara W.E., Marquez P., Thompson D., Su J.R., Licata C., Rosenblum H.G., Myers T.R. (2021). COVID-19 Vaccine Safety in Adolescents Aged 12–17 Years—United States, December 14, 2020–July 16, 2021. MMWR Morb. Mortal. Wkly. Rep..

[280] Leone F., Cerasuolo P.G., Bosello S.L., Verardi L., Fiori E., Cocciolillo F., Merlino B., Zoli A., D’Agostino M.A. (2021). Adult-Onset Still’s Disease Following COVID-19 Vaccination. Lancet Rheumatol..

[281] Rela M., Jothimani D., Vij M., Rajakumar A., Rammohan A. (2021). Auto-Immune Hepatitis Following COVID Vaccination. J. Autoimmun..

[282] Sebode M., Hartl J., Vergani D., Lohse A.W. (2018). The International Autoimmune Hepatitis Group (IAIHG) Autoimmune Hepatitis: From Current Knowledge and Clinical Practice to Future Research Agenda. Liver Int..

[283] Finsterer J. (2021). Guillain-Barre Syndrome 15 Days after COVID-19 despite SARS-CoV-2 Vaccination. IDCases.

[284] Thaler J., Ay C., Gleixner K.V., Hauswirth A.W., Cacioppo F., Grafeneder J., Quehenberger P., Pabinger I., Knöbl P. (2021). Successful Treatment of Vaccine-induced Prothrombotic Immune Thrombocytopenia (VIPIT). J. Thromb. Haemost..

[285] Cox D. (2021). Targeting SARS-CoV-2-Platelet Interactions in COVID-19 and Vaccine-Related Thrombosis. Front. Pharmacol..

[286] Cocco G., Delli Pizzi A., Fabiani S., Cocco N., Boccatonda A., Frisone A., Scarano A., Schiavone C. (2021). Lymphadenopathy after the Anti-COVID-19 Vaccine: Multiparametric Ultrasound Findings. Biology.

[287] Schiaffino S., Pinker K., Magni V., Cozzi A., Athanasiou A., Baltzer P.A.T., Camps Herrero J., Clauser P., Fallenberg E.M., Forrai G. (2021). Axillary Lymphadenopathy at the Time of COVID-19 Vaccination: Ten Recommendations from the European Society of Breast Imaging (EUSOBI). Insights Imaging.

[288] Dotan A., Shoenfeld Y. (2021). Perspectives on Vaccine Induced Thrombotic Thrombocytopenia. J. Autoimmun..

[289] Perumalswami P., Peng L., Odin J.A. (2009). Vaccination as a Triggering Event for Autoimmune Hepatitis. Semin. Liver Dis..

[290] van Gemeren M.A.J., van Wijngaarden P., Doukas M., de Man R.A. (2017). Vaccine-Related Autoimmune Hepatitis: The Same Disease as Idiopathic Autoimmune Hepatitis? Two Clinical Reports and Review. Scand. J. Gastroenterol..

[291] Asplund Högelin K., Ruffin N., Pin E., Månberg A., Hober S., Gafvelin G., Grönlund H., Nilsson P., Khademi M., Olsson T. (2021). Development of Humoral and Cellular Immunological Memory against SARS-CoV-2 despite B Cell Depleting Treatment in Multiple Sclerosis. iScience.

[292] Marinelli G., Inchingolo A.D., Inchingolo A.M., Malcangi G., Limongelli L., Montenegro V., Coloccia G., Laudadio C., Patano A., Inchingolo F. (2021). White Spot Lesions in Orthodontics: Prevention and Treatment. A Descriptive Review. J. Biol. Regul. Homeost. Agents.

[293] Adina S. (2020). Orthopedic Joint Stability Influences Growth and Maxillary Development: Clinical Aspects. J. Biol. Regul. Homeost. Agents.

[294] Patianna A.G., Ballini A., Meneghello M., Cantore S., Inchingolo A.M., Dipalma G., Inchingolo A.D., Inchingolo F., Malcangi G., Lucchese A. (2019). Comparison of Conventional Orthognathic Surgery and “Surgery-First” Protocol: A New Weapon against Time. J. Biol. Regul. Homeost Agents.

[295] Cantore S., Ballini A., De Vito D., Martelli F.S., Georgakopoulos I., Almasri M., Dibello V., Altini V., Farronato G., Dipalma G. (2017). Characterization of Human Apical Papilla-Derived Stem Cells. J. Biol. Regul. Homeost. Agents.

[296] Ballini A., Cantore S., Fotopoulou E.A., Georgakopoulos I.P., Athanasiou E., Bellos D., Paduanelli G., Saini R., Dipalma G., Inchingolo F. (2019). Combined Sea Salt-Based Oral Rinse with Xylitol in Orthodontic Patients: Clinical and Microbiological Study. J. Biol. Regul. Homeost. Agents.

[297] Cantore S., Ballini A., Farronato D., Malcangi G., Dipalma G., Assandri F., Garagiola U., Inchingolo F., De Vito D., Cirulli N. (2016). Evaluation of an Oral Appliance in Patients with Mild to Moderate Obstructive Sleep Apnea Syndrome Intolerant to Continuous Positive Airway Pressure Use: Preliminary Results. Int. J. Immunopathol. Pharm..

[298] Ballini A., Cantore S., Scacco S., Perillo L., Scarano A., Aityan S.K., Contaldo M., Cd Nguyen K., Santacroce L., Syed J. (2019). A Comparative Study on Different Stemness Gene Expression between Dental Pulp Stem Cells vs. Dental Bud Stem Cells. Eur. Rev. Med. Pharm. Sci..

[299] Iannetta M., Cesta N., Stingone C., Malagnino V., Teti E., Vitale P., De Simone G., Rossi B., Ansaldo L., Compagno M. (2020). Mild Clinical Manifestations of SARS-CoV-2 Related Pneumonia in Two Patients with Multiple Sclerosis under Treatment with Ocrelizumab. Mult. Scler. Relat. Disord..

[300] Khayat-Khoei M., Conway S., Rubinson D.A., Jarolim P., Houtchens M.K. (2021). Negative Anti-SARS-CoV-2 S Antibody Response Following Pfizer SARS-CoV-2 Vaccination in a Patient on Ocrelizumab. J. Neurol..

[301] Smets I., Reyes S., Baker D., Giovannoni G. (2021). Blunted Vaccines Responses after Ocrelizumab Highlight Need for Immunizations Prior to Treatment. Mult. Scler. Relat. Disord..

[302] Thornton J.R., Harel A. (2020). Negative SARS-CoV-2 Antibody Testing Following COVID-19 Infection in Two MS Patients Treated with Ocrelizumab. Mult. Scler. Relat. Disord..

[303] Achiron A., Mandel M., Dreyer-Alster S., Harari G., Magalashvili D., Sonis P., Dolev M., Menascu S., Flechter S., Falb R. (2021). Humoral Immune Response to COVID-19 MRNA Vaccine in Patients with Multiple Sclerosis Treated with High-Efficacy Disease-Modifying Therapies. Adv. Neurol. Disord..

[304] Murugesan K., Jagannathan P., Pham T.D., Pandey S., Bonilla H.F., Jacobson K., Parsonnet J., Andrews J.R., Weiskopf D., Sette A. (2021). Interferon-γ Release Assay for Accurate Detection of Severe Acute Respiratory Syndrome Coronavirus 2 T-Cell Response. Clin. Infect. Dis..

[305] Iannetta M., Landi D., Cola G., Malagnino V., Teti E., Fraboni D., Buccisano F., Grelli S., Coppola L., Campogiani L. (2021). T-Cell Responses to SARS-CoV-2 in Multiple Sclerosis Patients Treated with Ocrelizumab Healed from COVID-19 with Absent or Low Anti-Spike Antibody Titers. Mult. Scler. Relat. Disord..

[306] Bajaj V., Gadi N., Spihlman A.P., Wu S.C., Choi C.H., Moulton V.R. (2021). Aging, Immunity, and COVID-19: How Age Influences the Host Immune Response to Coronavirus Infections?. Front. Physiol..

[307] Wei J., Stoesser N., Matthews P.C., Ayoubkhani D., Studley R., Bell I., Bell J.I., Newton J.N., Farrar J., Diamond I. (2021). Antibody Responses to SARS-CoV-2 Vaccines in 45,965 Adults from the General Population of the United Kingdom. Nat. Microbiol..

[308] Yang H.S., Costa V., Racine-Brzostek S.E., Acker K.P., Yee J., Chen Z., Karbaschi M., Zuk R., Rand S., Sukhu A. (2021). Association of Age With SARS-CoV-2 Antibody Response. JAMA Netw. Open.

[309] García-Hernández M.H., Rodríguez-Varela E., García-Jacobo R.E., Hernández-De la Torre M., Uresti-Rivera E.E., González-Amaro R., Portales-Pérez D.P. (2018). Frequency of Regulatory B Cells in Adipose Tissue and Peripheral Blood from Individuals with Overweight, Obesity and Normal-Weight. Obes. Res. Clin. Pract..

[310] Sheridan P.A., Paich H.A., Handy J., Karlsson E.A., Hudgens M.G., Sammon A.B., Holland L.A., Weir S., Noah T.L., Beck M.A. (2012). Obesity Is Associated with Impaired Immune Response to Influenza Vaccination in Humans. Int. J. Obes..

[311] Eliakim A., Swindt C., Zaldivar F., Casali P., Cooper D.M. (2006). Reduced Tetanus Antibody Titers in Overweight Children. Autoimmunity.

[312] Lacson E., Argyropoulos C.P., Manley H.J., Aweh G., Chin A.I., Salman L.H., Hsu C.M., Johnson D.S., Weiner D.E. (2021). Immunogenicity of SARS-CoV-2 Vaccine in Dialysis. J. Am. Soc. Nephrol..

[313] Malard F., Gaugler B., Gozlan J., Bouquet L., Fofana D., Siblany L., Eshagh D., Adotevi O., Laheurte C., Ricard L. (2021). Weak Immunogenicity of SARS-CoV-2 Vaccine in Patients with Hematologic Malignancies. Blood Cancer J..

[314] Michiels Y., Houhou-Fidouh N., Collin G., Berger J., Kohli E. (2021). Impact of Low-Dose Methotrexate–Adalimumab Combination Therapy on the Antibody Response Induced by the MRNA-1273 SARS-CoV-2 Vaccine: Case of an Elderly Patient with Rheumatoid Arthritis. Vaccines.

[315] Anand S.P., Prévost J., Nayrac M., Beaudoin-Bussières G., Benlarbi M., Gasser R., Brassard N., Laumaea A., Gong S.Y., Bourassa C. (2021). Longitudinal Analysis of Humoral Immunity against SARS-CoV-2 Spike in Convalescent Individuals up to 8 Months Post-Symptom Onset. Cell Rep. Med..

[316] Tissot N., Brunel A.-S., Bozon F., Rosolen B., Chirouze C., Bouiller K. (2021). Patients with History of COVID-19 Had More Side Effects after the First Dose of COVID-19 Vaccine. Vaccine.

[317] Modenese A., Paduano S., Bargellini A., Bellucci R., Marchetti S., Bruno F., Grazioli P., Vivoli R., Gobba F. (2021). Neutralizing Anti-SARS-CoV-2 Antibody Titer and Reported Adverse Effects, in a Sample of Italian Nursing Home Personnel after Two Doses of the BNT162b2 Vaccine Administered Four Weeks Apart. Vaccines.

[318] Helmold Hait S., Vargas-Inchaustegui D.A., Musich T., Mohanram V., Tuero I., Venzon D.J., Bear J., Rosati M., Vaccari M., Franchini G. (2019). Early T Follicular Helper Cell Responses and Germinal Center Reactions Are Associated with Viremia Control in Immunized Rhesus Macaques. J. Virol..

[319] Turner J.S., O’Halloran J.A., Kalaidina E., Kim W., Schmitz A.J., Zhou J.Q., Lei T., Thapa M., Chen R.E., Case J.B. (2021). SARS-CoV-2 MRNA Vaccines Induce Persistent Human Germinal Centre Responses. Nature.

[320] Kaneko N., Kuo H.-H., Boucau J., Farmer J.R., Allard-Chamard H., Mahajan V.S., Piechocka-Trocha A., Lefteri K., Osborn M., Bals J. (2020). Loss of Bcl-6-Expressing T Follicular Helper Cells and Germinal Centers in COVID-19. Cell.

[321] Barrett J.R., Belij-Rammerstorfer S., Dold C., Ewer K.J., Folegatti P.M., Gilbride C., Halkerston R., Hill J., Jenkin D., Stockdale L. (2021). Phase 1/2 Trial of SARS-CoV-2 Vaccine ChAdOx1 NCoV-19 with a Booster Dose Induces Multifunctional Antibody Responses. Nat. Med..

[322] Khoury D.S., Cromer D., Reynaldi A., Schlub T.E., Wheatley A.K., Juno J.A., Subbarao K., Kent S.J., Triccas J.A., Davenport M.P. (2021). Neutralizing Antibody Levels Are Highly Predictive of Immune Protection from Symptomatic SARS-CoV-2 Infection. Nat. Med..

[323] Brochot E., Demey B., Touzé A., Belouzard S., Dubuisson J., Schmit J.-L., Duverlie G., Francois C., Castelain S., Helle F. (2020). Anti-Spike, Anti-Nucleocapsid and Neutralizing Antibodies in SARS-CoV-2 Inpatients and Asymptomatic Individuals. Front. Microbiol..

[324] Tauzin A., Nayrac M., Benlarbi M., Gong S.Y., Gasser R., Beaudoin-Bussières G., Brassard N., Laumaea A., Vézina D., Prévost J. (2021). A Single BNT162b2 MRNA Dose Elicits Antibodies with Fc-Mediated Effector Functions and Boost Pre-Existing Humoral and T Cell Responses. Immunology.

[325] Trougakos I.P., Terpos E., Zirou C., Sklirou A.D., Apostolakou F., Gumeni S., Charitaki I., Papanagnou E.-D., Bagratuni T., Liacos C.-I. (2021). Comparative Kinetics of SARS-CoV-2 Anti-Spike Protein RBD IgGs and Neutralizing Antibodies in Convalescent and Naïve Recipients of the BNT162b2 MRNA Vaccine versus COVID-19 Patients. BMC Med..

[326] Levi R., Azzolini E., Pozzi C., Ubaldi L., Lagioia M., Mantovani A., Rescigno M. (2021). One Dose of SARS-CoV-2 Vaccine Exponentially Increases Antibodies in Individuals Who Have Recovered from Symptomatic COVID-19. J. Clin. Investig..

[327] Doria-Rose N., Suthar M.S., Makowski M., O’Connell S., McDermott A.B., Flach B., Ledgerwood J.E., Mascola J.R., Graham B.S., Lin B.C. (2021). Antibody Persistence through 6 Months after the Second Dose of MRNA-1273 Vaccine for COVID-19. N. Engl. J. Med..

[328] Scarano A., Noumbissi S., Gupta S., Inchingolo F., Stilla P., Lorusso F. (2019). Scanning Electron Microscopy Analysis and Energy Dispersion X-Ray Microanalysis to Evaluate the Effects of Decontamination Chemicals and Heat Sterilization on Implant Surgical Drills: Zirconia vs. Steel. Appl. Sci..

[329] Scarano A., Piattelli A., Assenza B., Carinci F., Donato L.D., Romani GLMerla A. (2020). Infrared Thermographic Evaluation of Temperature Modifications Induced during Implant Site Preparation with Steel vs. Zirconia Implant Drill. J. Clin. Med..

[330] Scarano A., Murmura G., Vantaggiato G., Lauritano D., Silvestre-Rangil J., Di Cerbo A., Lorusso F. (2017). Delayed Expansion of Atrophic Mandible (Deam): A Case Report. Oral Implantol..

[331] Balzanelli M.G., Distratis P., Dipalma G., Vimercati L., Inchingolo A.D., Lazzaro R., Aityan S.K., Maggiore M.E., Mancini A., Laforgia R. (2021). SARS-CoV-2 Virus Infection May Interfere CD34+ Hematopoietic Stem Cells and Megakaryocyte–Erythroid Progenitors Differentiation Contributing to Platelet Defection towards Insurgence of Thrombocytopenia and Thrombophilia. Microorganisms.

[332] Rapone B., Ferrara E., Corsalini M., Qorri E., Converti I., Lorusso F., Delvecchio M., Gnoni A., Scacco S., Scarano A. (2021). Inflammatory Status and Glycemic Control Level of Patients with Type 2 Diabetes and Periodontitis: A Randomized Clinical Trial. Int. J. Environ. Res. Public Health.

[333] Kalemaj Z., Scarano A., Valbonetti L., Rapone B., Grassi F.R. (2016). Bone Response to Four Dental Implants with Different Surface Topographies: A Histologic and Histometric Study in Minipigs. Int. J. Periodontics Restor. Dent..

[334] Grassi F.R., Grassi R., Rapone B., Alemanno G., Balena A., Kalemaj Z. (2019). Dimensional Changes of Buccal Bone Plate in Immediate Implants Inserted through Open Flap, Open Flap and Bone Grafting and Flapless Techniques: A Cone-Beam Computed Tomography Randomized Controlled Clinical Trial. Clin. Oral Implants Res..

[335] Lorusso F., Noumbissi S., Francesco I., Rapone B., Khater A.G.A., Scarano A. (2020). Scientific Trends in Clinical Research on Zirconia Dental Implants: A Bibliometric Review. Materials.

[336] Favresse J., Bayart J.-L., Mullier F., Elsen M., Eucher C., Van Eeckhoudt S., Roy T., Wieers G., Laurent C., Dogné J.-M. (2021). Antibody Titres Decline 3-Month Post-Vaccination with BNT162b2. Emerg. Microbes Infect..

[337] Lorusso F., Tartaglia G., Inchingolo F., Scarano A. (2022). Early Response and Clinical Efficacy of a Mouthwash Containing Chlorhexidine, Anti Discoloration System, Polyvinylpyrrolidone/Vinyl Acetate and Sodium DNA in Periodontitis Model: A Triple-Blind Randomized Controlled Clinical Trial. Dent. J..

[338] Müller L., Andrée M., Moskorz W., Drexler I., Walotka L., Grothmann R., Ptok J., Hillebrandt J., Ritchie A., Rabl D. (2021). Age-Dependent Immune Response to the Biontech/Pfizer BNT162b2 Coronavirus Disease 2019 Vaccination. Clin. Infect. Dis..

[339] Macedo-Ojeda G., Muñoz-Valle J., Yokogawa-Teraoka P., Machado-Sulbarán A., Loza-Rojas M., Arredondo A.G., Tejeda-Constantini R., Vega-Magaña A., González-Estevez G., García-Chagollán M. (2021). COVID-19 Screening By Anti-SARS-CoV-2 Antibody Seropositivity: Clinical And Epidemiological Characteristics, Comorbidities, And Food Intake Quality. Int. J. Environ. Res. Public Health.

[340] Pellini R., Venuti A., Pimpinelli F., Abril E., Blandino G., Campo F., Conti L., De Virgilio A., De Marco F., Di Domenico E.G. (2021). Early Onset of SARS-CoV-2 Antibodies after First Dose of BNT162b2: Correlation with Age, Gender and BMI. Vaccines.

[341] Zhang J., McCullough P.A., Tecson K.M. (2020). Vitamin D Deficiency in Association with Endothelial Dysfunction: Implications for Patients WithCOVID-19. Rev. Cardiovasc. Med..

[342] Bellocchio L., Bordea I.R., Ballini A., Lorusso F., Hazballa D., Isacco C.G., Malcangi G., Inchingolo A.D., Dipalma G., Inchingolo F. (2020). Environmental Issues and Neurological Manifestations Associated with COVID-19 Pandemic: New Aspects of the Disease?. Int. J. Environ. Res. Public Health.

[343] Inchingolo F., Martelli F.S., Gargiulo Isacco C., Borsani E., Cantore S., Corcioli F., Boddi A., Nguyễn K.C.D., De Vito D., Aityan S.K. (2020). Chronic Periodontitis and Immunity, Towards the Implementation of a Personalized Medicine: A Translational Research on Gene Single Nucleotide Polymorphisms (SNPs) Linked to Chronic Oral Dysbiosis in 96 Caucasian Patients. Biomedicines.

[344] Ballini A., Gnoni A., De Vito D., Dipalma G., Cantore S., Gargiulo Isacco C., Saini R., Santacroce L., Topi S., Scarano A. (2019). Effect of Probiotics on the Occurrence of Nutrition Absorption Capacities in Healthy Children: A Randomized Double-Blinded Placebo-Controlled Pilot Study. Eur. Rev. Med. Pharm. Sci..

[345] Santacroce L., Inchingolo F., Topi S., Del Prete R., Di Cosola M., Charitos I.A., Montagnani M. (2021). Potential Beneficial Role of Probiotics on the Outcome of COVID-19 Patients: An Evolving Perspective. Diabetes Metab. Syndr. Clin. Res. Rev..

[346] Bastard P., Rosen L.B., Zhang Q., Michailidis E., Hoffmann H.-H., Zhang Y., Dorgham K., Philippot Q., Rosain J., Béziat V. (2020). Autoantibodies against Type I IFNs in Patients with Life-Threatening COVID-19. Science.

[347] Lazear H.M., Schoggins J.W., Diamond M.S. (2019). Shared and Distinct Functions of Type I and Type III Interferons. Immunity.

[348] Balzanelli M.G., Distratis P., Dipalma G., Vimercati L., Catucci O., Amatulli F., Cefalo A., Lazzaro R., Palazzo D., Aityan S.K. (2021). Immunity Profiling of COVID-19 Infection, Dynamic Variations of Lymphocyte Subsets, a Comparative Analysis on Four Different Groups. Microorganisms.

[349] Busnadiego I., Fernbach S., Pohl M.O., Karakus U., Huber M., Trkola A., Stertz S., Hale B.G. (2020). Antiviral Activity of Type I, II, and III Interferons Counterbalances ACE2 Inducibility and Restricts SARS-CoV-2. mBio.

[350] Zhang Y., Xiao M., Zhang S., Xia P., Cao W., Jiang W., Chen H., Ding X., Zhao H., Zhang H. (2020). Coagulopathy and Antiphospholipid Antibodies in Patients with COVID-19. N. Engl. J. Med..

[351] Zhou Y., Han T., Chen J., Hou C., Hua L., He S., Guo Y., Zhang S., Wang Y., Yuan J. (2020). Clinical and Autoimmune Characteristics of Severe and Critical Cases of COVID-19. Clin. Transl. Sci..

[352] Zuniga M., Gomes C., Carsons S.E., Bender M.T., Cotzia P., Miao Q.R., Lee D.C., Rodriguez A. (2021). Autoimmunity to Annexin A2 Predicts Mortality among Hospitalised COVID-19 Patients. Eur. Respir. J..

[353] Gomes C., Zuniga M., Crotty K.A., Qian K., Tovar N.C., Lin L.H., Argyropoulos K.V., Clancy R., Izmirly P., Buyon J. (2021). Autoimmune Anti-DNA and Anti-Phosphatidylserine Antibodies Predict Development of Severe COVID-19. Life Sci. Alliance.

[354] Schultze J.L., Aschenbrenner A.C. (2021). COVID-19 and the Human Innate Immune System. Cell.

[355] Combadière B. (2020). Immunité Adaptative Contre Le Virus SARS-CoV-2. Med. Sci..

[356] Goel R.R., Painter M.M., Apostolidis S.A., Mathew D., Meng W., Rosenfeld A.M., Lundgreen K.A., Reynaldi A., Khoury D.S., Pattekar A. (2021). MRNA Vaccination Induces Durable Immune Memory to SARS-CoV-2 with Continued Evolution to Variants of Concern. Science.

[357] Callow K.A., Parry H.F., Sergeant M., Tyrrell D.A.J. (1990). The Time Course of the Immune Response to Experimental Coronavirus Infection of Man. Epidemiol. Infect..

[358] Novelli G., Biancolella M., Mehrian-Shai R., Colona V.L., Brito A.F., Grubaugh N.D., Vasiliou V., Luzzatto L., Reichardt J.K.V. (2021). COVID-19 One Year into the Pandemic: From Genetics and Genomics to Therapy, Vaccination, and Policy. Hum. Genom..

[359] Zeberg H., Pääbo S. (2021). A Genomic Region Associated with Protection against Severe COVID-19 Is Inherited from Neandertals. Proc. Natl. Acad. Sci. USA.

[360] Thevarajan I., Nguyen T.H.O., Koutsakos M., Druce J., Caly L., van de Sandt C.E., Jia X., Nicholson S., Catton M., Cowie B. (2020). Breadth of Concomitant Immune Responses Prior to Patient Recovery: A Case Report of Non-Severe COVID-19. Nat. Med..

[361] Cunha L.L., Perazzio S.F., Azzi J., Cravedi P., Riella L.V. (2020). Remodeling of the Immune Response With Aging: Immunosenescence and Its Potential Impact on COVID-19 Immune Response. Front. Immunol..

[362] Williamson E.J., Walker A.J., Bhaskaran K., Bacon S., Bates C., Morton C.E., Curtis H.J., Mehrkar A., Evans D., Inglesby P. (2020). Factors Associated with COVID-19-Related Death Using OpenSAFELY. Nature.

[363] Koning R., Bastard P., Casanova J.-L., Brouwer M.C., van de Beek D., The Amsterdam U.M.C. COVID-19 Biobank Investigators (2021). Autoantibodies against Type I Interferons Are Associated with Multi-Organ Failure in COVID-19 Patients. Intensive Care Med..

[364] Zhou W., Wang W. (2021). Auto-Antibodies against Type I IFNs Are Associated with Severe COVID-19 Pneumonia. Sig. Transduct. Target.

[365] Liao M., Liu Y., Yuan J., Wen Y., Xu G., Zhao J., Cheng L., Li J., Wang X., Wang F. (2020). Single-Cell Landscape of Bronchoalveolar Immune Cells in Patients with COVID-19. Nat. Med..

[366] Rodrigues T.S., de Sá K.S.G., Ishimoto A.Y., Becerra A., Oliveira S., Almeida L., Gonçalves A.V., Perucello D.B., Andrade W.A., Castro R. (2021). Inflammasomes Are Activated in Response to SARS-CoV-2 Infection and Are Associated with COVID-19 Severity in Patients. J. Exp. Med..

[367] Colona V.L., Vasiliou V., Watt J., Novelli G., Reichardt J.K.V. (2021). Update on Human Genetic Susceptibility to COVID-19: Susceptibility to Virus and Response. Hum Genom..

[368] Netea M.G., Domínguez-Andrés J., Barreiro L.B., Chavakis T., Divangahi M., Fuchs E., Joosten L.A.B., van der Meer J.W.M., Mhlanga M.M., Mulder W.J.M. (2020). Defining Trained Immunity and Its Role in Health and Disease. Nat Rev Immunol.

[369] Ellinghaus D., Degenhardt F., Bujanda L., Buti M., Albillos A., Invernizzi P., Fernández J., Prati D., Baselli G., Severe COVID-19 GWAS Group (2020). Genomewide Association Study of Severe COVID-19 with Respiratory Failure. N. Engl. J. Med..

[370] Zeberg H., Pääbo S. (2020). The Major Genetic Risk Factor for Severe COVID-19 Is Inherited from Neanderthals. Nature.

[371] Villaescusa L., Zaragozá F., Gayo-Abeleira I., Zaragozá C. (2022). A New Approach to the Management of COVID-19. Antagonists of IL-6: Siltuximab. Adv. Ther..

[372] Sagris M., Theofilis P., Antonopoulos A.S., Oikonomou E., Tsioufis K., Tousoulis D. (2022). Genetic Predisposition and Inflammatory Inhibitors in COVID-19: Where Do We Stand?. Biomedicines.

